# MSH2 is not required for either maintenance of DNA methylation or repeat contraction at the *FMR1* locus in fragile X syndrome or the *FXN* locus in Friedreich’s ataxia

**DOI:** 10.1186/s13072-025-00588-4

**Published:** 2025-04-28

**Authors:** Jessalyn Grant-Bier, Kathryn Ruppert, Bruce Hayward, Karen Usdin, Daman Kumari

**Affiliations:** 1 Section on Gene Structure and Disease, Laboratory of Cell and Molecular Biology, National Institute of Diabetes and Digestive and Kidney Diseases, National Institutes of Health, Bethesda, MD 20892 USA; 2 Present address: Cellular and Molecular Biology Program, Perelman School of Medicine, University of Pennsylvania, Philadelphia, PA 19104 USA

**Keywords:** Repeat expansion, Mismatch repair, Fragile X syndrome, Friedreich’s ataxia, MSH2, DNA methylation, DNA damage

## Abstract

**Background:**

Repeat-induced epigenetic changes are observed in many repeat expansion disorders (REDs). These changes result in transcriptional deficits and/or silencing of the associated gene. MSH2, a mismatch repair protein that is required for repeat expansion in the REDs, has been implicated in the maintenance of DNA methylation seen in the region upstream of the expanded CTG repeats at the *DMPK* locus in myotonic dystrophy type 1 (DM1). Here, we investigated the role of MSH2 in aberrant DNA methylation in two additional REDs, fragile X syndrome (FXS) that is caused by a CGG repeat expansion in the 5’ untranslated region (UTR) of the *Fragile X Messenger Ribonucleoprotein 1* (*FMR1*) gene, and Friedreich’s ataxia (FRDA) that is caused by a GAA repeat expansion in intron 1 of the *frataxin* (*FXN*) gene.

**Results:**

In contrast to what is seen at the *DMPK* locus in DM1, loss of MSH2 did not decrease DNA methylation at the *FMR1* promoter in FXS embryonic stem cells (ESCs) or increase *FMR1* transcription. This difference was not due to the differences in the CpG density of the two loci as a decrease in DNA methylation was also not observed in a less CpG dense region upstream of the expanded GAA repeats in the *FXN* gene in MSH2 null induced pluripotent stem cells (iPSCs) derived from FRDA patient fibroblasts. Surprisingly, given previous reports, we found that *FMR1* reactivation was associated with a high frequency of MSH2-independent CGG-repeat contractions that resulted a permanent loss of DNA methylation. MSH2-independent GAA-repeat contractions were also seen in FRDA cells.

**Conclusions:**

Our results suggest that there are mechanistic differences in the way that DNA methylation is maintained in the region upstream of expanded repeats among different REDs even though they share a similar mechanism of repeat expansion. The high frequency of transcription-induced MSH2-dependent and MSH2-independent contractions we have observed may contribute to the mosaicism that is frequently seen in carriers of *FMR1* alleles with expanded CGG-repeat tracts. These contractions may reflect the underlying problems associated with transcription through the repeat. Given the recent interest in the therapeutic use of transcription-driven repeat contractions, our data may have interesting mechanistic, prognostic, and therapeutic implications.

**Graphical abstract:**

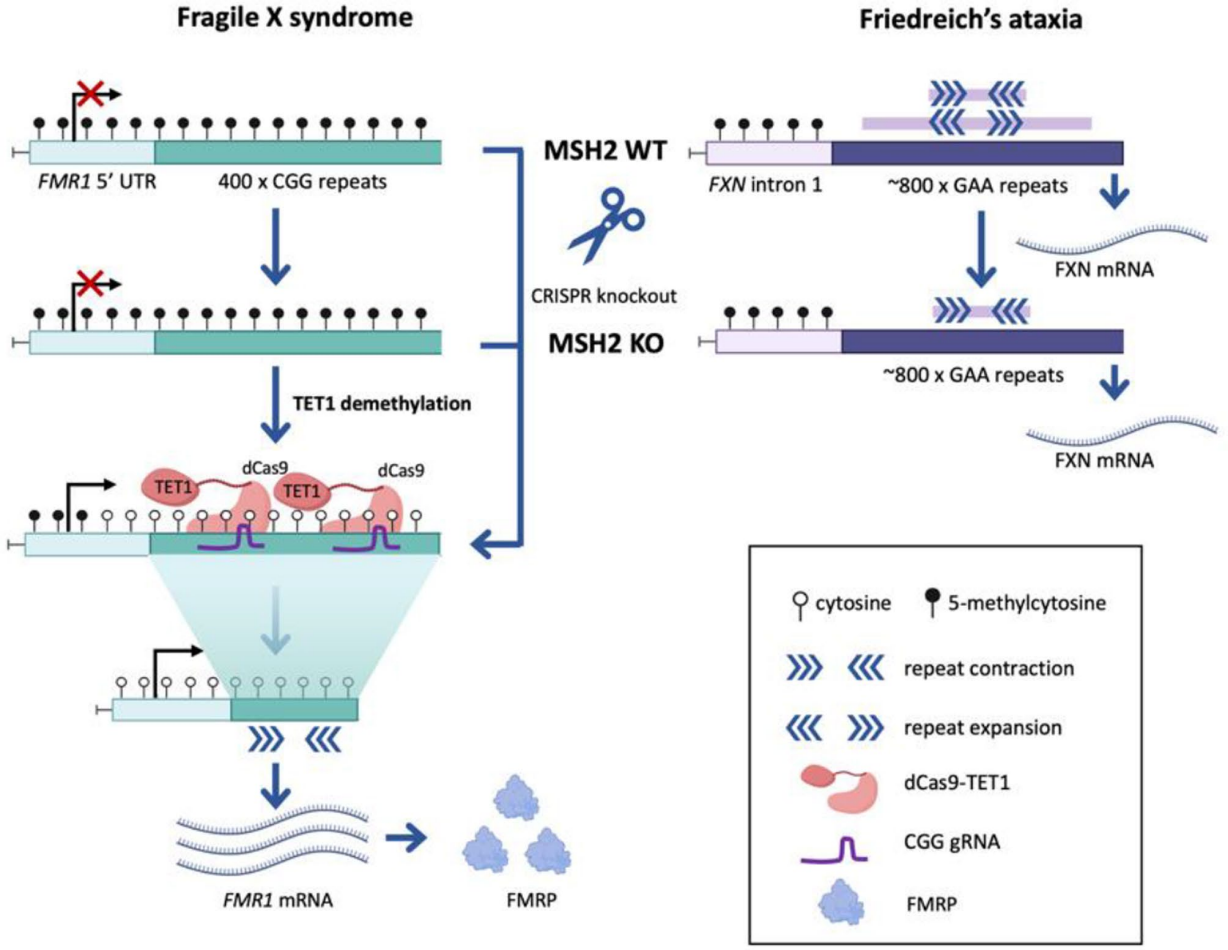

**Supplementary Information:**

The online version contains supplementary material available at 10.1186/s13072-025-00588-4.

## Background

There are more than 50 human genetic disorders that are all caused by the expansion of a short tandem repeat tract in a single specific gene [1]. Collectively, these are known as repeat expansion disorders (REDs). While the unit length, sequence and location of the repeat tract differ, all REDs share some common features. Many of the expanded repeats form one or more secondary structures [2, 3] that block DNA replication [4–8] and transcription [9–13]. Such structures are thought to contribute to the instability of the repeats as well as to some of the downstream consequences of repeat expansion including epigenetic changes that negatively affect transcription.

Fragile X syndrome (FXS; OMIM #300624), an X-linked neurodevelopmental disorder, is caused by the expansion of a CGG trinucleotide repeat in the 5’ untranslated region (UTR) of the *Fragile X Messenger Ribonucleoprotein 1* (*FMR1)* gene [14]. Typical *FMR1* alleles in unaffected individuals carry 6–54 CGG repeats. *FMR1* alleles with CGG repeats between 50 and 200 are known as premutation (PM) alleles. PM alleles make higher levels of *FMR1* mRNA than typical alleles, but reduced levels of encoded protein Fragile X Messenger Ribonucleoprotein (FMRP) due to the negative effect of expanded CGG repeats on translation [15–17]. When the CGG repeat expands beyond a threshold of 200 repeats, the 5’ end of the gene including the promoter and the repeats themselves, become hypermethylated resulting in transcriptional silencing [18, 19]. These alleles are known as full mutation (FM) alleles. Transcriptional silencing of FM alleles results in the absence of FMRP, an RNA binding protein important for synaptic plasticity [20] and results in intellectual disability. Repeat expansion-induced DNA methylation has also been reported in other REDs including Friedreich’s ataxia (FRDA) [21–23] and myotonic dystrophy type 1 (DM1) [24, 25]. FRDA (OMIM #229300) is an autosomal recessive progressive neurodegenerative disorder caused by the expansion of a GAA repeat in intron 1 of the *frataxin* (*FXN*) gene. Typical *FXN* alleles in unaffected individuals have between 7 and 33 GAA repeats and FRDA patients have between 66 and 1700 repeats [26, 27]. Repeat expansion causes a deficit of *FXN* mRNA by affecting both transcription initiation and elongation [10–12]. This results in reduced expression of frataxin, a ubiquitous and highly conserved mitochondrial protein [28]. Some of the CpG residues upstream of the expanded GAA repeats are hypermethylated in FRDA cells [21–23] with the extent of hypermethylation being directly related to GAA repeat size [23]. Increased methylation of CpG residues is also seen upstream of the expanded repeats in DM1 (OMIM #160900), an autosomal dominant disorder characterized by a CTG repeat expansion in the 3’ UTR of the *Dystrophy Myotonic Protein Kinase* (*DMPK)* gene [24, 25]. A typical *DMPK* allele has 5–37 CTG repeats whereas DM1 patients have between 50 and 5000 CTG repeats in the *DMPK* gene. The differentially methylated region (DMR) near the CTG repeats becomes hypermethylated when the repeat number exceeds 300 in human embryonic stem cells (hESCs) [29]. CTG repeat expansion affects the expression of *DMPK* and neighboring genes, *Sine Oculis Homeobox Homolog 5* (*SIX5*) and *Dystrophia Myotonica WD Repeat-Containing* (*DMWD*) which may contribute to the DM1 phenotype [30–33]. In addition to increased DNA methylation, *FMR1*, *FXN* and *DMPK* alleles with expanded repeats are all enriched for repressive histone modifications that include hypoacetylated histones and histone H3 di- and tri-methylated at lysine 9 (H3K9me2/3) [22, 34–37]. In addition, *FMR1* and *FXN* loci with expanded repeats are also enriched for histone H3 trimethylated at lysine 27 (H3K27me3) and histone H4 trimethylated at lysine 20 (H4K20me3) [37–39]. A better understanding of why and how repeat expansion causes these epigenetic changes may allow the development of interventions that could restore gene expression in affected REDs.

MutS homolog 2 (MSH2) is a protein that dimerizes with either MSH6 or MSH3 to form the mismatch repair (MMR) complexes MutSα and MutSβ respectively. MutSα binds to base mismatches and MutSβ to short insertion/deletion loops to initiate MMR [40]. In addition to its role in MMR and protection against microsatellite instability [41, 42], MSH2 is involved in other cellular processes important for maintaining genomic integrity including repair of double-strand breaks (DSBs) at stalled replication forks in human cells [43] and mitotic DNA synthesis to protect the stability of common fragile sites [44]. MSH2 is enriched at many REDs associated repeats [45–48] and transcription through the expanded repeats drives both expansions and contractions in an MSH2-dependent manner [49–54].

Recently, MSH2 was also shown to be involved in the maintenance of repeat-mediated DNA methylation in DM1 ESCs [55]. It has also been shown to be involved in the maintenance of transcriptional silencing of *DUX4* [56], a gene located within the D4Z4 macrosatellite repeat array in the sub-telomeric regions of the long arms of chromosomes 4 and 10 that is developmentally silenced by DNA methylation and H3K9me3 [57]. MSH2 has also been shown to recruit DNA methyltransferase 1 (DNMT1) to sites of oxidative damage [58]. The resulting DNA methylation has been suggested to cause a transient decrease in transcription to limit damage during the repair process, with the epigenetic state of the region being restored once DNA repair is completed [58]. However, persistent DNA damage has been shown to result in heritable gene silencing [59]. Loci with expanded CGG, GAA and CTG repeats are associated with increased DNA damage that causes repeat instability, and are prone to replication stress that has been shown to result in chromosome fragility [4, 7, 60–64].

Given the role of MSH2 in repeat instability in DM1, FXS and FRDA cells [65–68] and the propensity for increased DNA damage at *DMPK*, *FMR1* and *FXN* alleles with expanded repeats, it is possible that, as was seen with the *DMPK* locus in DM1, the maintenance of the repeat-induced DNA methylation at the *FMR1* and *FXN* loci may also be MSH2-dependent. To test this hypothesis, we used the CRISPR/Cas9 system to generate *MSH2* knockout (KO) cell lines from FXS ESCs with 400 CGG repeats in the *FMR1* gene and from induced pluripotent stem cells (iPSCs) derived from FRDA fibroblasts with ~ 800 GAA repeats in the *FXN* gene. We studied the role of MSH2 in maintaining DNA methylation in the *FMR1* gene promoter upstream of the CGG repeats and the intron 1 region upstream of the GAA repeats in the *FXN* gene. We focused on regions upstream of the expanded repeats because their methylation is directly and tightly correlated with *FMR1* gene silencing in FXS [19] and *FXN* mRNA deficit in FRDA [23]. We observed no difference in the CpG methylation in *MSH2* KO cell lines from both FXS ESCs and FRDA iPSCs after 2–3 months in culture. Our results indicate that in contrast to what was observed in DM1 cells, MSH2 does not play a role in maintaining DNA methylation at expanded FXS or FRDA alleles. In the course of doing this work, we also demonstrate that transcriptional activation of silenced *FMR1* alleles drives repeat contractions in FXS ESCs, many of which are MSH2-independent. These contractions may contribute to the repeat size mosaicism that is commonly seen in individuals who inherit *FMR1* alleles with large numbers of repeats. As such they may contribute to the variability in clinical presentation and disease penetrance that is observed.

## Methods

### Cell lines and culture conditions

Human male FXS ESCs (WCMC37) were obtained from Nikica Zaninovic, Weill Cornell Medical College of Cornell University (New York, NY). The isolation and characterization of the WCMC37F clone, that has one copy of a methylated *FMR1* gene with ~ 400 CGG repeats, has been previously described [69]. The FRDA iPSCs (GM23404), derived from a female individual, were obtained from Coriell Institute for Medical Research (Camden, NJ). Both alleles of the *FXN* gene have expanded GAA repeats in the 750–850 range in these FRDA iPSCs. FXS ESCs and FRDA iPSCs were cultured on tissue culture dishes coated with 5 µg/mL CellAdhere™ Laminin-521 (STEMCELL Technologies, Cambridge, MA, 200 − 0117) in StemFlex™ medium (Thermo Fisher Scientific, Waltham, MA, A3349401,) supplemented with 1X antibiotic-antimycotic (Thermo Fisher Scientific,15240096) at 37˚C in 5% CO_2_. Medium was changed every other day. Cell passaging was performed twice a week using StemPro™ Accutase™ (Thermo Fisher Scientific, A11105-01) and cells were plated in growth medium supplemented with RevitaCell™ supplement (Thermo Fisher Scientific, A2644501). Total RNA from H1 ESCs (WA01, WiCell Research Institute, Madison, WI) carrying typical *FMR1* alleles with 30 CGG repeats was used as a control for measuring *FMR1* mRNA levels and genomic DNA from GM06865 (Coriell Institute for Medical Research) lymphoblastoid cells, that carry typical *FMR1* alleles with 30 CGG repeats, was used as a control in the bisulfite sequencing for *FMR1* promoter methylation analysis. Genomic DNA from a human iPSC line carrying typical *FXN* alleles with 10 GAA repeats was used as a control in the bisulfite sequencing for methylation analysis of the *FXN* locus.

### Generation of *MSH2* knockout cell lines using CRISPR/nCas9

Genome edits were made using nCas9 (D10A nickase mutant) to create a single-stranded break at two locations in the *MSH2* gene within exon 3. Repair by non-homologous end joining (NHEJ) resulted in random mutations, especially large > 40 bp deletions, which led to complete knockout of MSH2. The nCas9 sgRNAs were previously described [55] and shown to have high editing efficiency in hESCs. To generate a fragment containing two complete sgRNA expression cassettes, oligonucleotide primers pX462-MSH2-dual-F and pX462-MSH2-dual-R (Table S1) were annealed to a U6-gRNA scaffold template containing gRNA-scaffold followed by U6 promoter as previously described [70]. The PCR was done using Q5 Hot Start High-Fidelity DNA Polymerase (New England Biolabs, Ipswich, MA, M0493L) to generate a fragment that was cloned into a BbsI digested pSpCas9n(BB)-2 A-Puro (PX462) V2.0, a gift from Feng Zhang (Addgene plasmid 62987; http://n2t.net/addgene:62987) [71] using the NEB HiFi DNA Assembly Cloning kit (New England Biolabs, E2621L). The ligated DNA was transformed into DH5alpha competent cells (New England Biolabs, C2987I) and plasmid DNA was isolated using the Monarch miniprep kit (New England Biolabs, T1010L). Vector assembly for pX462-MSH2-dual-gRNA was verified by Sanger sequencing (Psomagen, Rockville, MD), and transfection-grade plasmid DNA was prepared using the NucleoBond Xtra Midi Plus kit (Macherey-Nagel, Allentown, PA, 740422.5). Whole plasmid sequence is available in Supplementary Material 3.

FXS ESCs and FRDA iPSCs (1.7 × 10^5/ well) were plated in 12-well plates coated with Laminin-521 one day before transfection to be 50% confluent at time of transfection. Cells were transfected with pX462-MSH2-dual-gRNA and pCE-mp53DD, a gift from Shinya Yamanaka (Addgene plasmid # 41856; http://n2t.net/addgene:41856) [72] using Lipofectamine STEM (Thermo Fisher Scientific, STEM00008) according to the manufacturer’s guidelines. pCE-mp53DD was used to improve cell survival after CRISPR editing. Twenty-four hours after transfection, 1 µg/mL puromycin (Thermo Fisher Scientific, A11138-03) was added for 48 h to select for transfected cells. After puromycin selection, cells were grown until well-separated colonies were visible and then passaged as single cells at low densities on 60 mm dishes coated with Laminin-521 and grown until colonies formed. Colonies were scraped and replated individually into 24 well plates. FXS ESCs and FRDA iPSCs were also transfected with only pCE-mp53DD plasmid and single-cell colonies were picked to serve as MSH2 wild type (WT) controls.

### Sequence validation of the *MSH2* knockout

Exon 3 of the *MSH2* gene was PCR amplified using Q5^®^ Hot Start High-Fidelity DNA Polymerase with the following conditions: 98˚C for 1 min, (98˚C for 15 s, 59˚C for 15 s, 72˚C for 40 s) x 35 cycles, and a final elongation step at 72˚C for 2 min using 1 µM of Gb_MSH2_3F and Gb_MSH2_3R2 primers (Table S1). PCR products were either cleaned using ExoSAP-IT reagent (Thermo Fisher Scientific, 78201.1ML) or purified using NEB Monarch PCR cleanup kit. Cleaned PCR products were Sanger sequenced (Psomagen) and analyzed using the Synthego ICE software (Synthego Performance Analysis, ICE Analysis. 2019. v3.0. Synthego; [Accessed March 15, 2023]) to rule out the presence of a WT MSH2 exon 3 allele.

### Western blot analysis

To prepare total cell lysates, 1–2 × 10^6^ cells were washed once with cold PBS and resuspended in lysis buffer containing 10 mM Tris pH 7.5, 1mM EDTA pH 8.0, 1% Triton X-100 (Sigma-Aldrich, St. Louis, MO, T8787-50ML), 1X protease inhibitor cocktail (Millipore Sigma, Burlington, MA, P8340-5ML), and 1X phosphatase inhibitor cocktail (Sigma-Aldrich, P5726-1ML). After 10 min incubation on ice, lysate was sonicated in Bioruptor (Diagenode, Denville, NJ) on medium setting for 1 min, 30s on/30s off and stored at -80˚C. Protein concentrations were measured using the Bradford Protein Assay Dye Reagent (Bio-Rad Laboratories, Hercules, CA, 5000006), and then 1X LDS loading buffer (Thermo Fisher Scientific, NP0007) and 1X Reducing Agent (Thermo Fisher Scientific, NP0009) were added to the lysate and heated for 5 min at 95˚C. For MSH2 detection, a total of 20 µg lysate was run on a NuPAGE™ 4–12% Bis-Tris Gel (Thermo Fisher Scientific, NP0322BOX) in 1X MOPS SDS Running buffer (Thermo Fisher Scientific, NP0001) at 200 V for 45 min. Proteins were transferred onto a nitrocellulose membrane using the Trans-Blot Turbo RTA transfer kit (Bio-Rad Laboratories, 1704270) and Trans-Blot Turbo Transfer system at 25 V for 14 min. Nitrocellulose membranes were blocked for 1 h with 5% Amersham ECL Prime Blocking reagent (Millipore-Sigma, RPN418) in 1X TBST (Thermo Fisher Scientific, 28360). Blots were incubated overnight at 4˚C in 1:10,000 diluted rabbit anti-human MSH2 antibody (Abcam, Waltham, MA, ab70270). The next day, blots were washed 3 times with 1X TBST for 5 min each and incubated for 1 h at room temperature with 1:5000 diluted secondary anti-rabbit HRP conjugated antibody (Sigma Aldrich, GENA934-1ML). Blots were washed 3 times in TBST for 5 min each, and once in 1X TBS, and then detected with ECL prime western blot detection reagent (Millipore-Sigma, GERPN2232) for 5 min in dark. Bands were visualized using the Chemidoc Imaging System (Bio-Rad Laboratories). Blots were washed with 1X TBS and incubated with 1:1000 diluted anti-β-actin antibody (Abcam, ab8227) overnight at 4˚C. The blot was then processed and imaged as before. For FMRP detection, 10 µg of total cell lysate was run on a NuPAGE™ 4–12% Bis-Tris Gel and transferred to nitrocellulose membrane as described above. The blot was incubated with 1:2000 dilution of anti-FMRP antibody (BioLegend, 834601) overnight at 4˚C followed by three washes and incubation with 1:2000 diluted secondary anti-mouse HRP conjugated antibody (Millipore Sigma, 12–249) for 1 h at RT, washed and detected with ECL prime western blot detection reagent and visualized using the Chemidoc Imaging System. Blot was then washed and probed with 1:10,000 diluted β-actin monoclonal antibody (Thermo Fisher Scientific, MA1-140) as a loading control, washed and incubated with 1:5000 diluted secondary anti-mouse HRP conjugated antibody for 45 min at RT, washed and detected with ECL prime western blot detection reagent and visualized using the Chemidoc Imaging System. Quantitation was done using the Image Lab software (BioRad).

### Immunofluorescence

CRISPR edited FXS MSH2 KO-2 and FXS MSH2 WT-2 cells were plated in 24 well plates (2 × 10^5^ cells per well). The next day, cells were washed with PBS, fixed with 4% formaldehyde at 4 °C for 45 min and washed three times with PBS. Cells were blocked in blocking buffer [PBS containing 10% normal goat serum (Thermo Fisher Scientific, PCN5000) and 0.3% Triton X-100 (Sigma-Aldrich, T8787-50ML)] for 1 h. Fixed cells were then incubated with 1:1000 rabbit anti-human MSH2 antibody (Abcam, ab70270) diluted in PBS containing 1% BSA (Millipore Sigma, A7030-10G), 1% normal goat serum and 0.3% Triton X-100 at 4 °C overnight. After three washes with 0.1% BSA in PBS, cells were incubated with 1:2000 secondary antibody (Thermo Fisher Scientific, A-11034) diluted in PBS containing 1% BSA, 1% normal goat serum and 0.3% Triton X-100 for 45 min. Nuclei were stained with DAPI (Thermo Fisher Scientific, 66248) diluted to 0.25 ug/mL in PBS with 0.1% BSA. For FMRP detection, 1:500 dilution of anti-FMRP antibody (BioLegend, 834601) was used followed by 1:2000 Alexa Fluor™ 555 labeled anti-mouse IgG (H + L) highly cross-adsorbed secondary antibody (Thermo Fisher Scientific, A-21424). Images were taken on BioTek Cytation 5 Cell Imaging Multimode Reader (Agilent, Santa Clara, CA) and processed with Fiji software (version 2.14.0/1.54f) [73]. FMRP intensity in each cell was quantified using BioTek Gen5 software (version 3.15, Agilent).

### RNA methods

Total RNA was isolated from cells using TRIzol™ reagent (Thermo Fisher Scientific) according to manufacturer’s instructions. RNA was quantified using DS-11 (DeNovix, Wilmington, DE). Three hundred nanograms of RNA was reverse transcribed to cDNA in 20 µL final volume using SuperScript™ IV VILO™ master mix (Thermo Fisher Scientific, 11756050) as per manufacturer’s instructions. Real-time PCR was performed in triplicate using 2 µL of the cDNA, FAM-labeled *FMR1* (Hs00924547_m1) and VIC-labeled *b-glucuronidase* (*GUSB*) endogenous control (4326320E) Taqman probe-primers (Thermo Fisher Scientific) and TaqMan Fast Advanced master mix (Thermo Fisher Scientific, 4444964) on QuantStudio 3 Real-Time PCR system (Thermo Fisher Scientific). For quantitation, the comparative threshold (ddCt) method was used.

### Analysis of CGG and GAA repeats via PCR

Genomic DNA was isolated by the salting out method [74]. For *FMR1* CGG repeat PCR, 1 µg of genomic DNA from FXS cells was digested overnight at 37 °C in a total reaction volume of 50 µl in rCutSmart Buffer (New England Biolabs, B6004S) with either only HindIII-HF (New England Biolabs, R3104S) or HindIII-HF and HpaII (New England Biolabs, R0171S). PCR was performed using 5 µL digested DNA (100 ng) in a 50 µL reaction containing 0.8 µM each of Not_FraxC and FAM-Not_FraxR4 primers (Table S1), 1X pH 9.0 Triton Buffer (50 mM Tris pH9, 1.5 mM MgCl2, 20 mM (NH_4_)_2_SO_4_, 0.2% Triton X-100), 2.8 M betaine, 2.3% DMSO, 0.6 mM dNTPs and 0.5 units of Q5 Hot Start High-Fidelity DNA Polymerase using the following thermocycler conditions: 98 °C for 3 min, (98 °C for 30 s, 65 °C for 30 s, 72 °C for 3 min 30 s) x 33 cycles, and a final elongation step at 72 °C for 10 min. For *FXN* GAA repeat PCR, ~ 100 ng genomic DNA was amplified using 0.5 µM each of GAA-104 F and GAA-629R primers (Table S1) in a 20 µL reaction with 1X Q5 buffer, 0.2 mM dNTPs and 0.4 units Q5^®^ Hot Start High-Fidelity DNA Polymerase with following thermocycler conditions: 98 °C for 1 min, (98 °C for 15 s, 70 °C for 15 s, 72 °C for 3 min) x 35 cycles, and a final elongation step at 72 °C for 10 min. For small pool *FXN* GAA repeat PCR, 1 ng of genomic DNA (~ 150 genomes) was used per reaction using the same protocol. For small pool *FMR1* CGG repeat PCR, a 200 µL master mix was assembled with 0.5 µM each of Not_FraxC and FAM-Not_FraxR4 primers (Table S1), 1X pH 9.0 Triton Buffer (50 mM Tris pH9, 1.5 mM MgCl2, 20 mM (NH_4_)_2_SO_4_, 0.2% Triton X-100), 2.5 M betaine, 2% DMSO, 0.5 mM dNTPs, 4 units of Q5 Hot Start High-Fidelity DNA Polymerase and 20 ng of genomic DNA. The 100 µl master mix was then divided into 10 µl aliquots each containing ~ 300 genomes and PCR performed using the following thermocycler conditions: 98 °C for 3 min, (98 °C for 30 s, 65 °C for 30 s, 72 °C for 3 min 30 s) x 35 cycles, and a final elongation step at 72 °C for 10 min. Products were run on a 1% TAE agarose gel in 1X TAE buffer.

### Methylation-specific qPCR

To analyze DNA methylation at the *FMR1* promoter, methylation-specific qPCR was performed as previously described [69, 75]. A total of 1 µg genomic DNA was diluted to 10 ng/µL in TE pH 8.0 and sonicated in Bioruptor for 5 min at medium setting, 30 s on/30 seconds off. To confirm that the fragment size was less that 1 kb, 15 µL of DNA was run on a 1% agarose gel in 1X TAE buffer. The sonicated DNA was divided into two tubes and was digested in one tube with HpaII restriction enzyme (New England Biolabs, R0171S) in rCutSmart Buffer (New England Biolabs, B6004S) overnight at 37˚C. The second tube of undigested DNA was also incubated in parallel. The samples were incubated at 80 °C for 20 min to inactivate HpaII. Real-time PCR was performed in triplicate using 2 µL each of undigested and digested DNAs using PowerUp™ SYBR™ Green Master mix (Thermo Fisher Scientific, A25777) using QuantStudio™ 3 Real-Time PCR machine. The *FMR1* promoter was amplified using primer pair *FMR1* ex1 F and ex1 R, and *GAPDH* was amplified using primer pair *GAPDH* exon1 F and *GAPDH* intron1 R (Table S1). *GAPDH* was used as an unmethylated control region to confirm HpaII digestion. For quantitation, the comparative threshold (ddCt) method was used. Ct values of digested (cut) samples were compared to undigested (uncut) samples to determine methylation levels. These were expressed as fold change (FC) of cut over uncut. The extent of methylation determined by this assay (FC over uncut) sometimes exceeds the value of 1 for methylated samples due to small differences in the PCR efficiency of undigested (uncut) and digested (cut) DNAs [75].

### Bisulfite conversion and sequencing

For the FXS samples, a total of 500 ng of genomic DNA was bisulfite-converted using the EZ DNA Methylation-Lightning Kit (Zymo Research, Irvine, CA, P212121) and eluted in 20 µL elution buffer. A hemi-nested PCR was performed to amplify the *FMR1* promoter region. For the outer PCR, 4 µL bisulfite converted DNA was added to a PCR master mix to final concentrations of 0.25 µM each for primer (FMR1-Met-1 F and Gb_Metbis-2645-R) (Table S1), 0.167 mM dNTPs, 1X Platinum II PCR buffer, and 0.04 U/µL Platinum™ II Taq Hot-Start DNA Polymerase (Thermo Fisher Scientific, 14966001). The PCR was performed by following cycles: 94 °C for 3 min, (94 °C for 30 s, 50 °C for 30 s, 72 °C for 1 min) x 20, and 72 °C for 4 min. The product was diluted 1:10 for the inner PCR. The inner PCR uses same PCR conditions except with Gb_FMR1-Met-2 F (Table S1) as the forward primer and 30 cycles.

For the FRDA samples, 2 µg of genomic DNA were digested for 3 h with HindIII restriction enzyme (New England Biolabs, R0104S) and stored overnight at -20 °C. Then, 200 ng of DNA was enzymatically bisulfite converted using the NEBNext^®^ Enzymatic Methyl-seq Conversion Kit (New England Biolabs, E7125S) which produced 100 µL of converted DNA at 2 ng/µL concentration. The bisulfite modified DNA was stored overnight at 4˚C. Then, 4 µL of DNA was used for PCR using 1.5 µM primers Gb_FXNMe-1212-F and Gb_ FXNMe_1930-R (Table S1 [22]),, 0.2 mM dNTPs, 1X Platinum II PCR Buffer and 0.04 U/µL Platinum™ II Taq Hot-Start DNA Polymerase. The PCR was performed by following cycles: 95 °C for 5 min, (95 °C for 30 s, 59 °C for 30 s, 72 °C for 2 min) x 35, and 72 °C for 10 min. The PCR products were cleaned using Zymo Select-a-Size DNA Clean and Concentrate (Zymo Research, D4084) and eluted in 20 µL NEB Elution Buffer (New England Biolabs, T1016L).

After determining the DNA concentration using the DS-11, bisulfite/enzymatic converted DNA samples were diluted to 7.5 ng/µL and analyzed by Plasmidsaurus Premium PCR Sequencing (https://plasmidsaurus.com/home) which uses Oxford Nanopore sequencing with an R10.4.1 flow cell. It generated 500–3000 individual reads that were aligned using NCBI BLAST software [76] to a bisulfite converted genomic DNA as template and analyzed using a custom Python3 script (Supplementary Material 1) to determine the methylation status of the 37 individual CpGs (for *FMR1*) and 17 CpGs (for *FXN*) and average methylation of the bulk population. Sequences that did not align or completely span the PCR amplified region were discarded.

### Transient transfection of dCas9-TET1 for *FMR1* reactivation

Gibson assembly [77] was used to construct the plasmid pMLM3636-Puro-dCas9-TET1 using NEBuilder^®^HiFi DNA Assembly Master Mix (New England Biolabs, E2621L) and fragments derived from the following plasmids: PuroR from pSpCas9(BB)-2 A-Puro (PX459) V2.0, a gift from Feng Zhang (Addgene plasmid # 62988; http://n2t.net/addgene:62988) [71], dCas9 from Inducible Caspex expression, a gift from Steven Carr & Samuel Myers (Addgene plasmid # 97421; http://n2t.net/addgene:97421) [78], and TET1CD from pPlatTET-gRNA2, a gift from Izuho Hatada (Addgene plasmid # 82559; http://n2t.net/addgene:82559) [79] and vector backbone from pMLM3636 (a gift from Keith Joung (Addgene plasmid # 43860; http://n2t.net/addgene:43860). Two additional c-MYC nuclear localization signal sequences (NLSs) were added using Gb_dCas9-cmyc-F (Table S1) to increase nuclear localization of dCas9-TET1. Various PCR fragments were purified with 0.5 volumes of Zymo Select-a-Size DNA Clean and Concentrator Magbead Kit (Zymo Research, # D4084) before assembly. The assembled DNA was transformed into DH5alpha competent cells (New England Biolabs, C2987I) and plasmid DNA was isolated using the Monarch miniprep kit (New England Biolabs, T1010L). Vector assembly was verified using Whole Plasmid Sequencing (Psomagen, Rockville, MD) and transfection-grade plasmid DNA was prepared using the NucleoBond Xtra Midi Plus kit (Macherey-Nagel, Allentown, PA, 740422.5).

To generate pMLM3636-Puro-dCas9-TET1-CGG, a GG(CGG)6 gRNA (gRNA-CGG_6_, Table S1) was cloned into pMLM3636-Puro-dCas9-TET1. To generate pMLM3636-Puro-dCas9-TET1-PRM with two different gRNAs in the promoter region (gRNA-PRM-1 and gRNA-PRM-2) previously described in [80], oligonucleotide primers PuroR-FMR1-gRNA-PRM_F and PuroR-FMR1-gRNA-PRM_R (Table S1) were annealed to a U6-gRNA scaffold template containing gRNA-scaffold followed by U6 promoter. The PCR was done using Q5 Hot Start High-Fidelity DNA Polymerase (New England Biolabs, Ipswich, MA, M0493L) to generate a fragment that was cloned into pMLM3636-Puro-dCas9-TET1. Whole plasmid sequences are provided in Supplementary Material 2, 4 and 5.

To transiently demethylate and reactivate the *FMR1* gene, FXS MSH2 WT-2 and FXS MSH2 KO-1 ESCs (8 × 10^5^/ well) were plated in 6-well plates one day before transfection to be 80% confluent at time of transfection. Cells were transfected with 1 µg of pMLM3636-Puro_dCas9-TET1-CGG6 and 200 ng of pCE-mp53DD using Lipofectamine STEM according to the manufacturer’s guidelines. Twenty-four hours after transfection, 1 µg/mL puromycin was added for 48 h to select for transfected cells. Surviving cells were grown for 40 days under the cell culture conditions described above in one well of a 6-well plate and DNA and RNA were collected every 7 days for analysis. For FMRP detection using western blot and immunofluorescence, FXS MSH2 WT-2 and FXS MSH2 KO-1 ESCs were transfected with plasmids pMLM3636-Puro_dCas9-TET1-CGG6 and pCE-mp53DD, puromycin selected for 48 h and grown for 8 days at which time they were passaged to collect DNA, RNA and lysate and plated in 24 well plate for immunofluorescence.

### PCR to detect plasmid presence in transfected cells

The presence of transfected plasmid in cells at different days post transfection was tested by PCR. Either 200 ng of genomic DNA isolated from transfected cells, or 1 ng of plasmid (used as a positive control) was used in the PCR reaction along with 1X Q5 buffer, 0.04 mM dNTPs, 0.5 μm each of primers 3636–727 F and 459-377R (Table S1) and 0.4 units Q5^®^ Hot Start High-Fidelity DNA Polymerase. The following thermocycler conditions were used: 98 °C for 1 min, (98 °C for 15 s, 60 °C for 30 s, 72 °C for 30 s) x 33 cycles, and a final elongation step at 72 °C for 2 min. The PCR products were run on a 1% TAE agarose gel in 1X TAE buffer.

### Statistical methods

The statistical significance of the differences between two samples was calculated by unpaired student’s t-test using a cutoff of *p* < 0.05 to determine significance. Determination of differential changes in CpG methylation over time between MSH2 WT and MSH2 KO cell lines was carried out using linear regression analysis with the R function *lm* from the *stats* package (v4.4.2). The model used percent methylation as the response variable and month (m1, m2, m3) and genotype (WT, KO) as the predictor variables, including their interaction term. Estimated p-values were corrected for multiple testing with the R function p.adjust (*stats* package). The Mann-Whitney U test was used to calculate the significance of difference in the intensity of FMRP expression in FXS MSH2 WT and FXS MSH2 KO cells after transfection with dCas9-TET1-CGG.

## Results

### Loss of MSH2 does not affect DNA methylation at the *FMR1* promoter in FXS ESCs

**Fig. 1:**
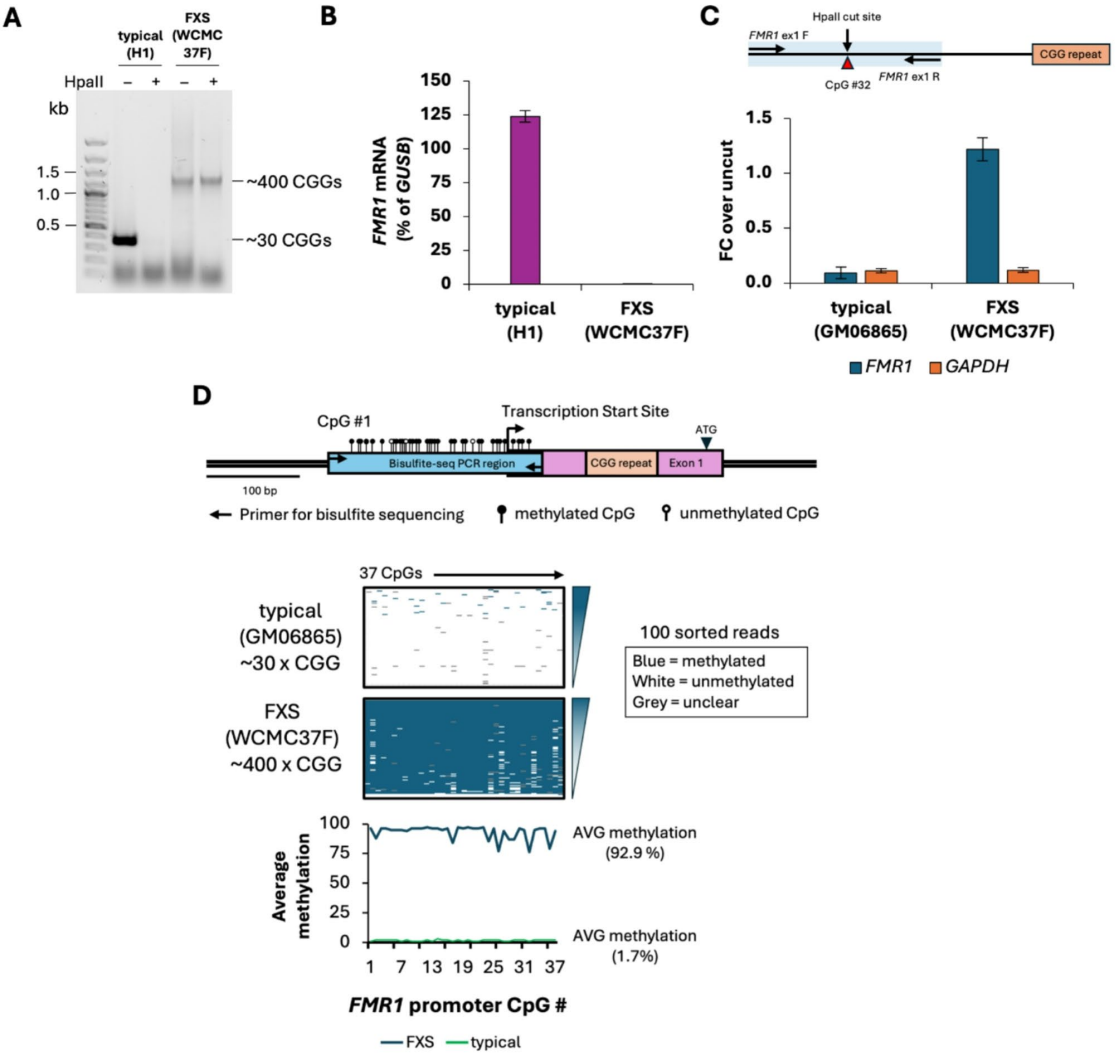
**(A)** Bulk CGG-repeat PCR with and without predigestion of genomic DNA with methylation sensitive HpaII in H1 (hESCs, male) that have a typical *FMR1* allele with 30 CGG repeats and WCMC37F (FXS ESCs, male) that have a methylated *FMR1* allele with ~ 400 CGG repeats. **(B)***FMR1* mRNA levels are shown as a percentage of *GUSB* mRNA in H1 ESCs, and FXS ESCs (WCMC37F). Data shown are an average from two replicate experiments. Error bars represent standard deviation. **(C)** Above, schematic showing the location of qPCR primers and methylation-sensitive HpaII restriction enzyme site in the *FMR1* promoter region analyzed by the methylation qPCR assay. Below, methylation qPCR in H1 and FXS ESCs. DNA methylation is shown as fold change (FC) over uncut. Data shown are an average of two replicates per cell line, and error bars represent standard deviation. *GAPDH* is used as an unmethylated control for HpaII digestion. **(D)** Above, schematic indicating location of the 37 CpG residues studied by bisulfite sequencing in the region 5’ of the CGG repeat in the *FMR1* promoter. Below, bisulfite sequencing data for individual CpG residues is shown from 100 randomly sorted individual DNA reads arranged vertically from most to least methylated per DNA sample from a typical cell line with 30 CGG repeats (GM06865) used as an assay control, and FXS ESCs (WCMC37F). Summary panel below shows the average methylation at individual CpGs in the *FMR1* promoter region

We confirmed the CGG repeat size, promoter methylation and the absence of *FMR1* transcription in FXS ESCs (WCMC37F, male) before making the edits to the *MSH2* locus (Fig. 1). The WCMC37F cells carry a single copy of a methylated *FMR1* allele with ~ 400 CGG repeats as assessed by methylation sensitive CGG repeat PCR (Fig. 1A). While H1 ESCs (male) carrying a typical *FMR1* allele with 30 CGG repeats (Fig. 1A) showed *FMR1* mRNA expression that was 120% of *GUSB* expression, *FMR1* mRNA levels in FXS ESCs were only 0.05% of *GUSB* (Fig. 1B). The *FMR1* promoter in FXS ESCs was fully methylated as evidenced by a methylation-sensitive qPCR assay which provides a quantitative measure of bulk methylation at HpaII sites in the *FMR1* promoter (Fig. 1C). We also performed bisulfite sequencing to look at the methylation status of 37 individual CpG sites in the *FMR1* promoter region from − 394 to − 93 bp upstream of the CGG repeat. After bisulfite conversion of DNA, long-read sequencing (Oxford Nanopore Technology) was used to determine the methylation profiles of 500–3000 individual DNA molecules for each line. Minor variations in the methylation of CpGs across different alleles in the sample were observed in cells with a typical *FMR1* allele with 30 CGG repeats as well as in the parental FXS ESC clone WCMC37F with ~ 400 CGG repeats. As expected, in typical alleles with 30 CGG repeats, there was very little methylation seen in the *FMR1* promoter region whereas in FX alleles with ~ 400 CGG repeats the average methylation of the region was > 90% and only a very small number of fully unmethylated alleles were observed (Fig. 1D). In fact, most alleles were either highly methylated (> 90%) or highly unmethylated (< 10%). This is consistent with the suggestion that methylation and demethylation are progressive processes that, once initiated, lead to complete methylation or demethylation [81]. Since long CGG repeats are notoriously difficult to amplify because they are 100% GC and prone to form secondary structures, unmethylated alleles which would have had many of their CGs residues converted to UG after bisulfite modification, tend to be preferentially amplified. Thus, the unmethylated alleles are likely to be overrepresented in this analysis.

**Fig. 2:**
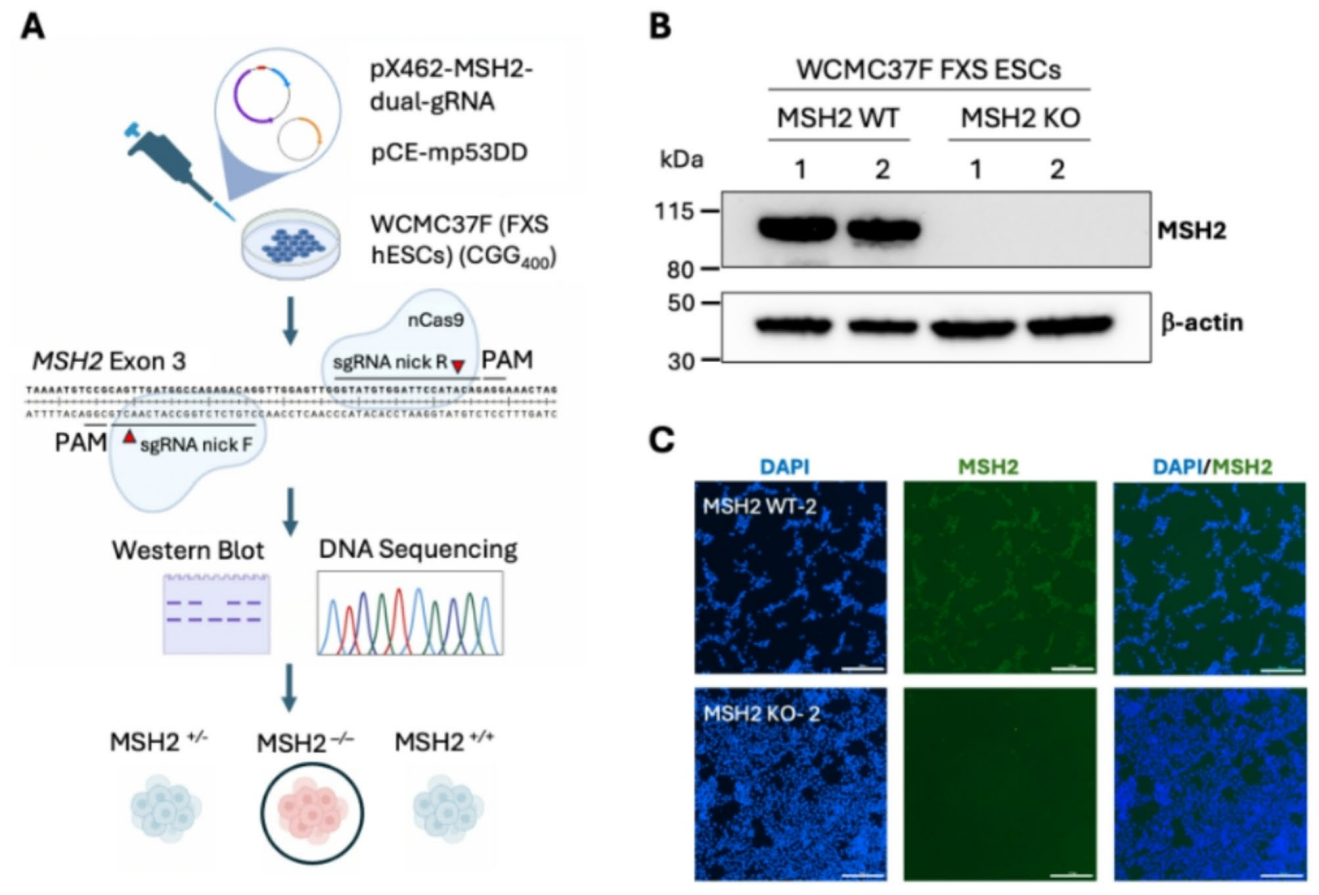
**(A)** A schematic showing the creation of *MSH2* null fragile X syndrome human embryonic stem cell lines (FXS hESCs). FXS ESCs (WCMC37F) were transfected with plasmids expressing nCas9 and dual guide RNAs targeting *MSH2* exon 3. Edited clones were screened using western blot and DNA sequencing, and *MSH2* null clones were selected for further study. **(B)** Western blot showing MSH2 expression in two cell lines each for FXS MSH2 WT and FXS MSH2 KO. β-actin is used as a loading control. Western blot of all clones assayed is shown in Figure S1B. **(C)** Immunofluorescence imaging for MSH2 (green) shows a strong, nuclear signal in FXS MSH2 WT-2 and the absence of MSH2 in FXS MSH2 KO-2 cells. Scale bars represent 300 μm

We used CRISPR/Cas9 genome editing with dual Cas9 nickases and 2 guide RNAs targeting human *MSH2* exon 3 to generate MSH2 KO FXS ESCs (Fig. 2A). A plasmid carrying a dominant negative version of p53, pCE-mp53DD, was co-transfected to increase cell survival. Exon 3 of *MSH2* encodes the DNA binding domain of the MSH2 protein, and the guide RNAs were chosen based on previous studies showing they generated efficient edits in hESCs [55]. To generate MSH2 WT control lines, FXS ESCs were transfected with pCE-mp53DD alone. To confirm the crispants, we sequenced a 631 bp region containing all of *MSH2* exon 3 and analyzed the mutations in both FXS MSH2 KO-1 and FXS MSH2 KO-2 cells using Synthego ICE software. We found complete loss of the parental allele in both cell lines caused by either large in-frame deletions or frameshift mutations (Figure S1A). The control cell lines, FXS MSH2 WT-1 and FXS MSH2 WT-2 showed no changes in the *MSH2* exon 3 sequence (Figure S1A). The levels of MSH2 in edited lines were analyzed using a western blot (Figure S1B) and 2 independent clones that showed a complete loss of MSH2 were selected for further studies (FXS MSH2 KO-1 and FXS MSH2 KO-2). Similarly, two clones from FXS ESCs transfected with pCE-mp53DD alone were selected as MSH2 WT control cell lines (FXS MSH2 WT-1 and FXS MSH2 WT-2). FXS MSH2 KO-1 and FXS MSH2 KO-2 showed no MSH2 expression when compared to FXS MSH2 WT-1 and FXS MSH2 WT-2 (Fig. 2B). Finally, we performed immunofluorescence for MSH2 and confirmed that MSH2 has strong nuclear expression in all FXS MSH2 WT-2 cells, while there was no detectable MSH2 expression in the crispant FXS MSH2 KO-2 cells (Fig. 2C).

**Fig. 3:**
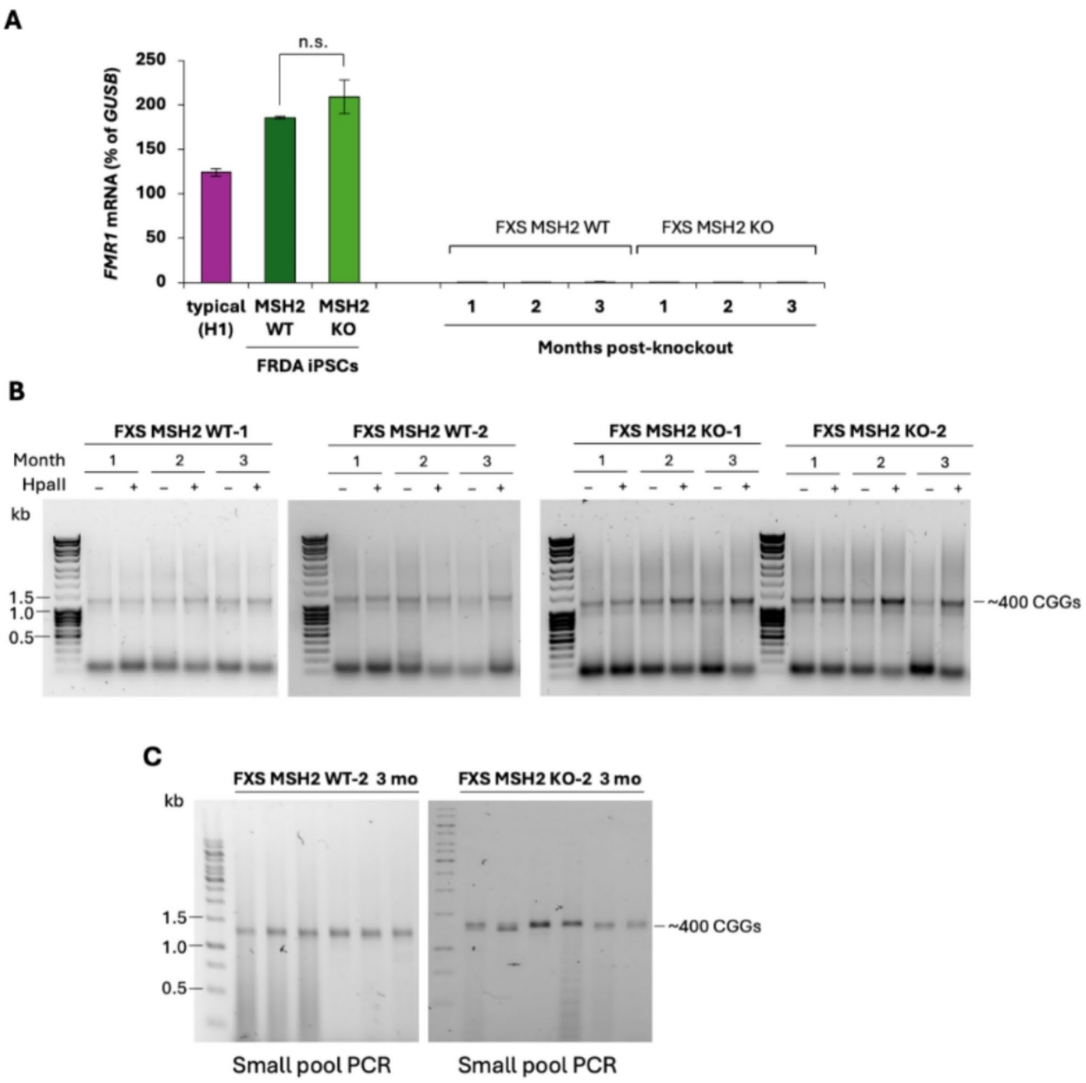
**(A)***FMR1* mRNA levels are shown as a percentage of *GUSB* mRNA in H1 ESCs carrying a typical *FMR1* allele with 30 CGG repeats, FRDA MSH2 WT and FRDA MSH2 KO iPSCs carrying typical *FMR1* alleles with 30 CGG repeats, and FXS MSH2 WT and FXS MSH2 KO ESCs carrying a FX allele with 400 CGG repeats. Data shown are an average from two replicates for H1 ESCs. For FRDA iPSCs, the data shown are an average of two replicates from two individual iPSCs lines each for MSH2 WT and MSH2 KO. For FXS ESCs, data shown are an average of two individual lines each for MSH2 WT and MSH2 KO. Error bars represent standard deviation. n.s., not significant. **(B)** Bulk CGG-repeat PCR in two individual cell lines each for FXS MSH2 WT and FXS MSH2 KO is shown at indicated times in culture with and without predigestion of genomic DNA with methylation-sensitive HpaII. 1 month (approximately 40–50 days), 2 months (approximately 70–80 days) and 3 months (approximately 100–110 days). **(C)** Small pool CGG repeat PCR for FXS MSH2 WT-2 and FXS MSH2 KO-2 lines at 3 months

The two MSH2 WT and two MSH2 KO FXS ESC lines were grown for at least 100 days in culture and *FMR1* mRNA levels, DNA methylation, and CGG repeat size were analyzed at indicated times for each line (Fig. 3). We first analyzed the *FMR1* mRNA levels as a proxy for any subtle decrease in DNA methylation. There was no significant change in the *FMR1* mRNA levels, with or without MSH2, in cell lines carrying typical *FMR1* alleles with 30 CGG repeats (Fig. 3A, Figure S2). The MSH2 WT and MSH2 KO FXS ESCs expressed very low levels of *FMR1* mRNA that did not change over 3 months in culture (Fig. 3A). The FXS MSH2 KO cell lines also did not show any obvious change in repeat size or DNA methylation when assessed by methylation-sensitive CGG repeat PCR (Fig. 3B) or by small pool CGG repeat PCR (Fig. 3C) over 3 months in culture.

**Fig. 4:**
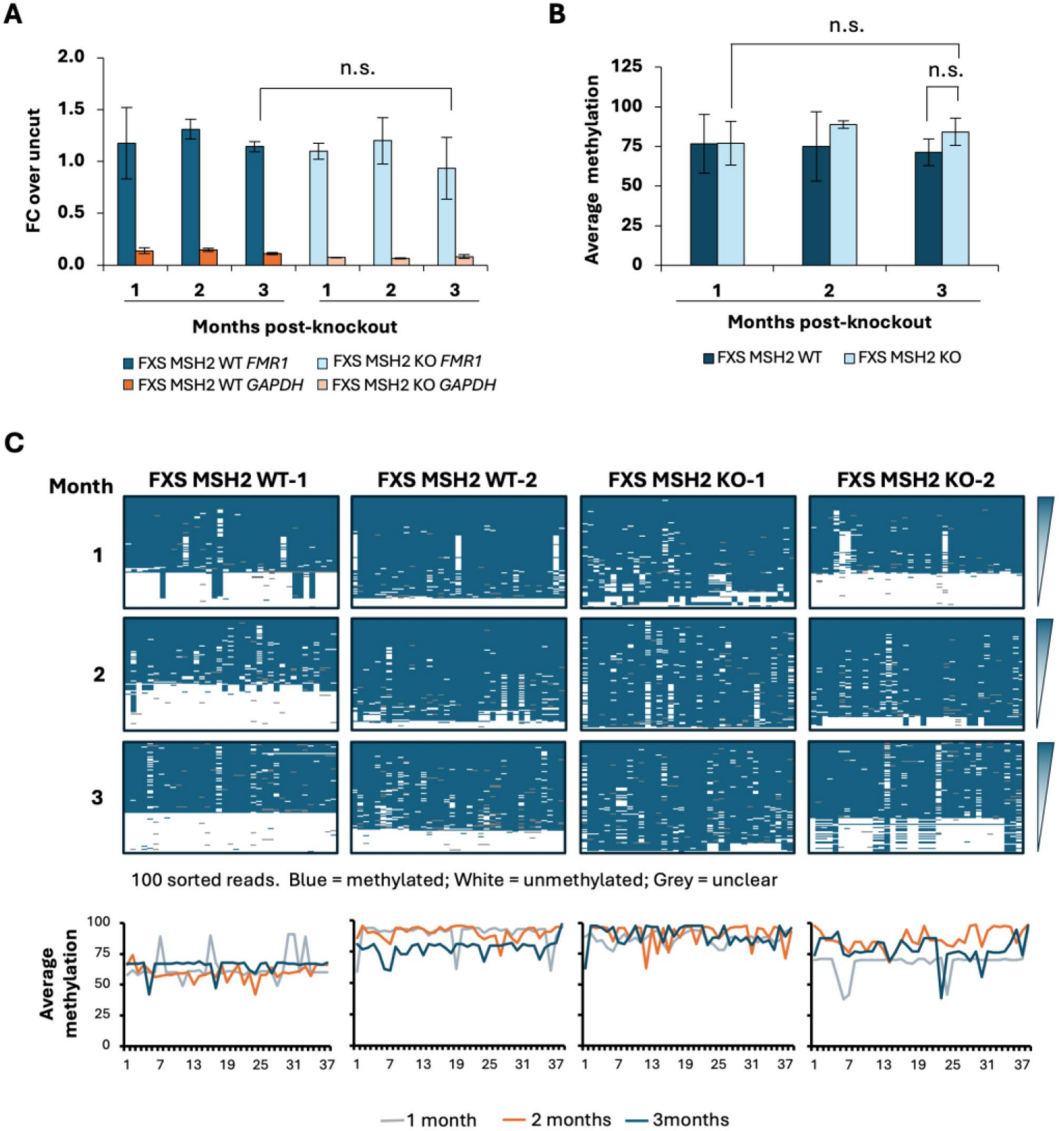
DNA methylation in FXS MSH2 WT and FXS MSH2 KO cell lines. **(A)** Methylation qPCR in FXS MSH2 WT and FXS MSH2 KO ESCs. DNA methylation data are shown as fold change (FC) over uncut and are an average from two cell lines each for FXS MSH2 WT and FXS MSH2 KO, and error bars represent standard deviation. *GAPDH* is used as an unmethylated control for HpaII digestion. n.s., not significant. **(B)** Average methylation in the *FMR1* promoter is shown for FXS MSH2 WT and FXS MSH2 KO cell lines at 1, 2 and 3 months. Bisulfite sequencing data for individual CpGs were averaged to compare overall methylation. Data shown is an average from 2 cell lines and error bars represent standard deviation. n.s., not significant. **(C)** Bisulfite sequencing data for individual CpG residues is shown from 100 randomly sorted individual DNA reads arranged vertically from most to least methylated per DNA sample for 1, 2 and 3 months. Summary panels show the comparison of average methylation per CpG over time in each cell line

Next, we analyzed the methylation levels in the *FMR1* promoter for the two MSH2 WT and two MSH2 KO cell lines using a methylation-specific qPCR assay. There was no significant difference in methylation between the FXS MSH2 KO and FXS MSH2 WT cell lines, indicating no change in the bulk methylation of the *FMR1* promoter more than 100 days post-MSH2 knockout (*p* = 0.75, Fig. 4A). Thus, the loss of MSH2 did not have any significant effect on overall DNA methylation levels at the *FMR1* promoter in FXS ESCs.

In DM1 ESCs, it was observed that specific CpG residues in the *DMPK* locus became demethylated after 2 months in culture post-MSH2 knockdown, while other CpGs remained methylated [55]. To test if MSH2 might have a similar effect on specific CpG residues in FXS ESCs, we performed bisulfite sequencing to look at the methylation status of 37 individual CpG sites in the *FMR1* promoter region from − 394 to − 93 bp upstream of the CGG repeat. There was a small increase in the average methylation of *FMR1* alleles in the FXS MSH2 KO cell lines over time; however, this difference was not statistically significant (*p* = 0.61) (Fig. 4B). Similarly, some *FMR1* alleles in FXS MSH2 WT lines were unmethylated to begin with, and there was a slight decrease in average methylation over time (Fig. 4B and C and Figure S3). However, there was no significant difference in the overall methylation levels between the FXS MSH2 WT lines and the FXS MSH2 KO lines at 3 months post-knockout (*p* = 0.27, Fig. 4B). Differential methylation analysis between MSH2 WT and MSH2 KO cells revealed no significant changes in methylation levels over time at CpG sites (adj. p-value > 0.05, Table S2). While a greater proportion of unmethylated alleles were detected in one MSH2 WT line (FXS MSH2 WT-1) and one MSH2 KO line (FXS MSH2 KO-2) (Fig. 4C and Figure S3), only very low levels of *FMR1* mRNA were detected (Fig. 3A) confirming that the *FMR1* gene is transcriptionally silenced in a majority of cells in the population. These unmethylated alleles may arise from spontaneous contractions of the fully methylated alleles. As discussed previously, the proportion of unmethylated alleles is likely to be overrepresented in the assay. In any event, since the fraction of such alleles detected did not change over the 3-month period, it suggests that no progressive demethylation has occurred in either set of cell lines.

### Loss of MSH2 does not affect DNA methylation at the *frataxin* locus in FRDA iPSCs

**Fig. 5:**
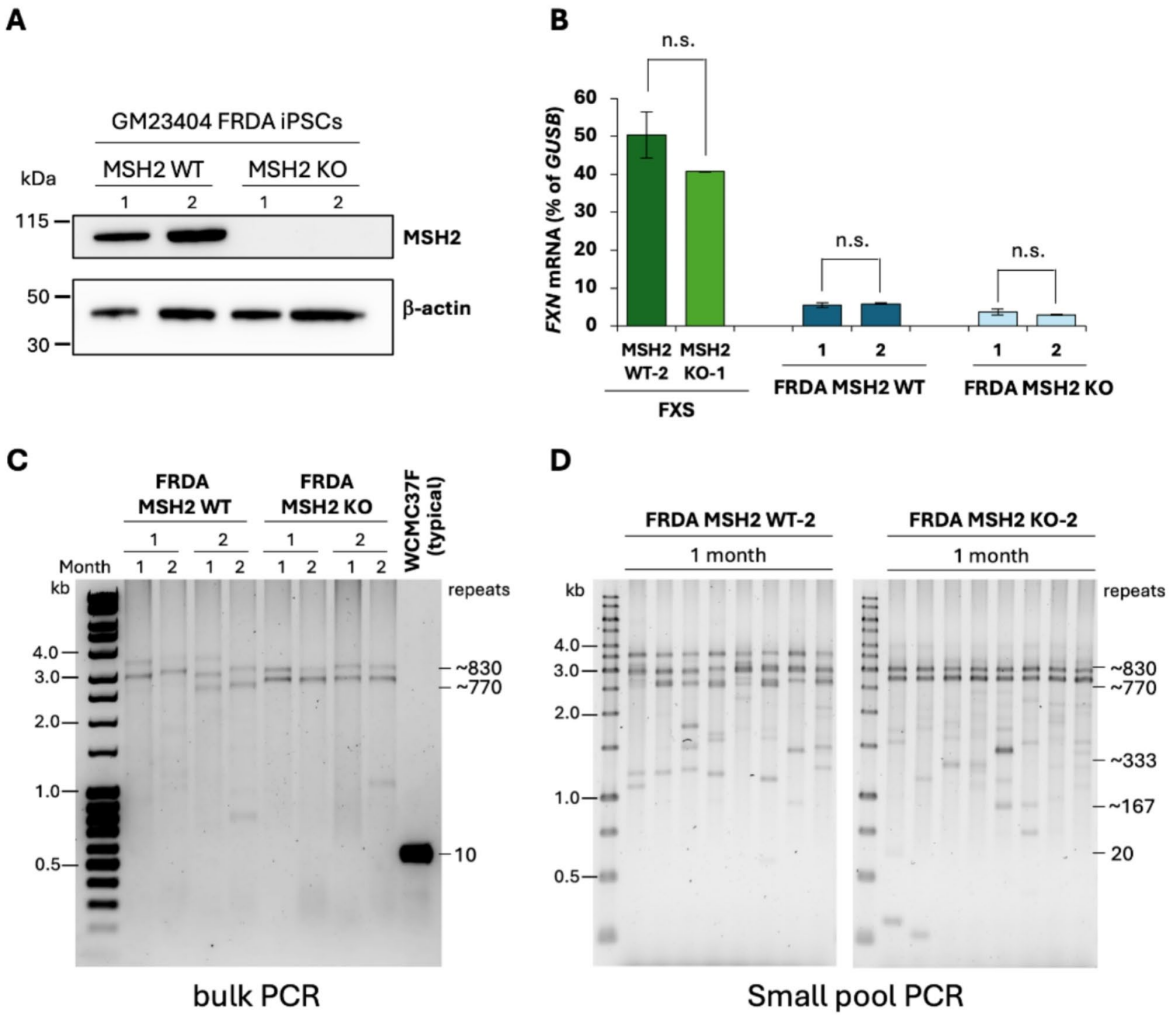
Validation of MSH2 CRISPR knockout in FRDA iPSCs (GM23404, female). **(A)** Western blot showing MSH2 expression in FRDA MSH2 WT and its loss in MSH2 KO cell lines. β-actin is used as a loading control. **(B)** Average *FXN* mRNA levels are shown as a percentage of *GUSB* mRNA from FXS MSH2 WT-2 and FXS MSH2 KO-1 cell lines carrying typical *FXN* alleles with 10 GAA repeats and FRDA MSH2 WT-1 and WT-2 and FRDA MSH2 KO-1 and KO-2 lines measured at 2 months post knockout. Data shown are an average from two independent experiments and the error bars represent standard deviation. n.s., not significant. **(C)** Bulk PCR for GAA repeat size for FRDA MSH2 WT-1 and WT-2, and FRDA MSH2 KO-1 and KO-2 cells at 1 and 2 months in culture. Genomic DNA from WCMC37F cells which carry *FXN* alleles with ~ 10 GAA repeats was used as a positive control (typical). **(D)** Small pool GAA repeat PCR results are shown from FRDA MSH2 WT-2 and FRDA MSH2 KO-2 at 1 month

In the *DMPK* gene, there are 25 CpG residues in the 318 bp region directly upstream of the CTG repeat, while the *FMR1* gene has 37 CpGs in the 349 bp region upstream of the CGG repeat (Figure S4A). However, the CGG repeats themselves contain 1 CpG per repeat, thus adding another 400 CpGs to the *FMR1* locus in the FXS ESCs used in the current study. To test whether the differences observed in the levels of DNA methylation upon loss of MSH2 between *DMPK* alleles in DM1 ESCs and *FMR1* alleles in FXS ESCs could be attributed to the differences in the overall CpG density, we decided to study the role of MSH2 in another repeat expansion disorder, FRDA, because the differentially methylated region upstream of expanded GAA repeat in *FXN* alleles is less CpG-rich (Figure S4) and the GAA repeat itself cannot be methylated. We generated two *MSH2* knockout clones (FRDA MSH2 KO-1 and FRDA MSH2 KO-2) and two mock-transfected control clones (FRDA MSH2 WT-1 and FRDA MSH2 WT-2) in an FRDA iPSC line (GM23404), which has ~ 750–850 GAA repeats in the *FXN* gene. We used the same dual nickase strategy and guide RNAs used to generate the *MSH2* knockout in FXS ESCs (Fig. 2A). We sequenced *MSH2* exon 3 PCR products from the FRDA MSH2 KO clones, analyzing them using Synthego ICE software (Figure S5A). We found that both FRDA MSH2 KO cell lines had complete loss of MSH2 due to large insertions or deletions. We further confirmed the loss of MSH2 via western blot (Fig. 5A and Figure S5B).

The MSH2 WT and MSH2 KO FRDA iPSC lines were grown for at least two months under the same conditions as the FXS ESCs. We measured *FXN* mRNA levels over time in the four FRDA iPSC lines. However, there was no consistent trend over time, and no significant difference in *FXN* mRNA levels was seen between FRDA MSH2 WT and FRDA MSH2 KO cell lines at two months post-transfection (*p* = 0.55, Fig. 5B). Loss of MSH2 did not affect *FXN* mRNA levels in FXS ESCs that carry typical *FXN* alleles with 10 GAA repeats (Fig. 5B and C).

To further characterize the lines, we performed GAA repeat PCR. Each cell line had slightly different GAA repeat sizes, but all were within the range of 700–850 repeats, reflecting the variability in repeat size in the parental population (Fig. 5C). We observed that over two months in culture, the FRDA MSH2 WT-1 and FRDA MSH2 WT-2 lines showed visible expansion: for each cell line, both alleles increased in size between one month and two months post mock-transfection. In FRDA MSH2 KO-1 and FRDA MSH2 KO-2, there was no increase in allele size over two months post-*MSH2* knockout. This is consistent with previous studies that showed that MSH2 is required for repeat expansion in FRDA iPSCs [68]. In both FRDA MSH2 WT and FRDA MSH2 KO cell lines, the GAA repeat PCR showed bands indicating stochastic contractions within the population. This had been previously observed in FRDA iPSC lines, but interestingly, while loss of MSH2 was previously reported to decrease contractions [68], our data show that many contractions were still seen in the absence of MSH2 (Fig. 5D).

**Fig. 6:**
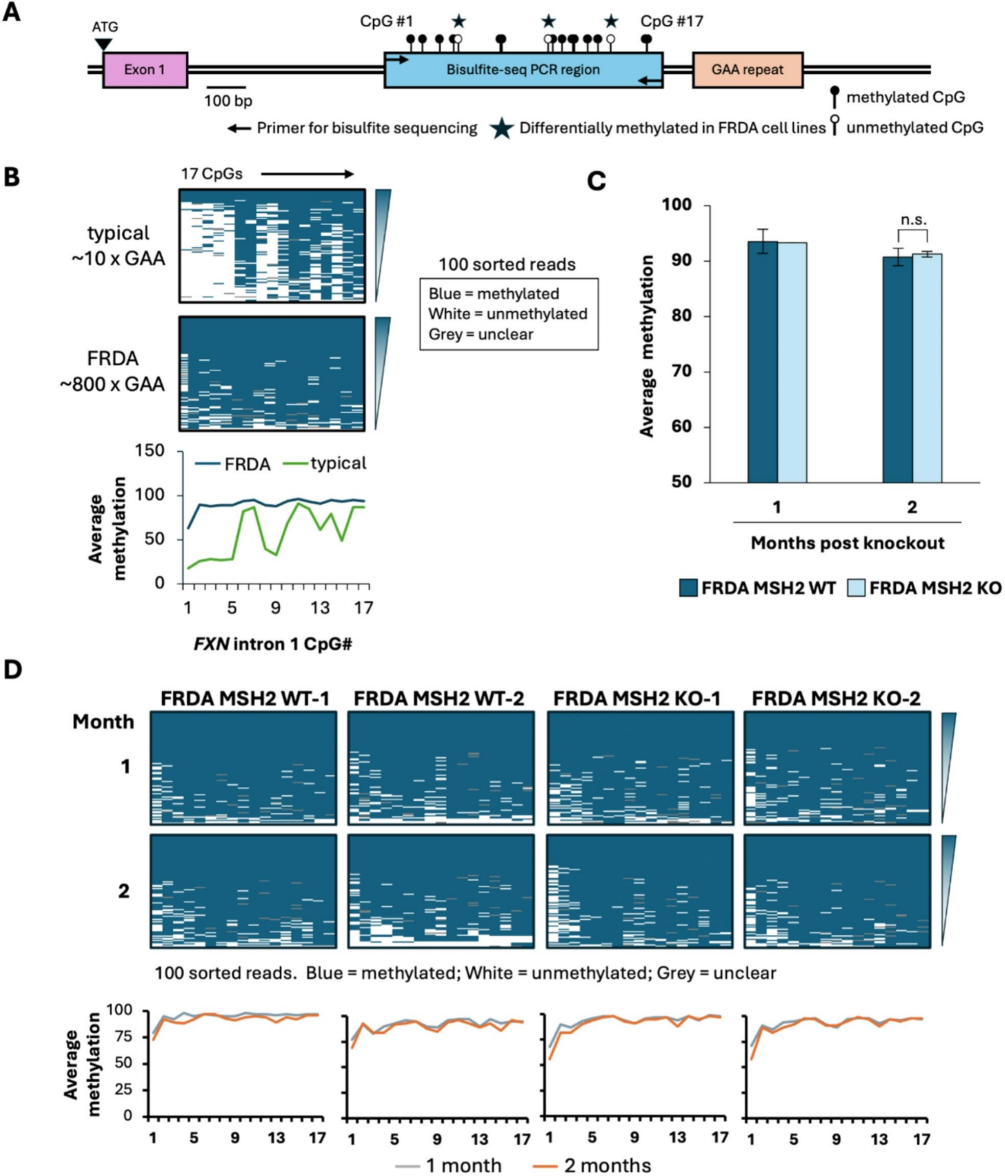
Bisulfite sequencing results for FRDA MSH2 WT and FRDA MSH2 KO cells. **(A)** Schematic indicating locations of the 17 CpG residues studied by bisulfite sequencing in the region 5’ of the GAA repeat in *FXN* intron 1. Residues marked with stars were found to be differentially methylated in FRDA cells compared to cells with typical *FXN* alleles. **(B)** Bisulfite sequencing results showing methylation at 17 CpGs residues in parental FRDA iPSCs (GM23404) carrying *FXN* alleles with ~ 800 GAA repeats and a human iPSC line carrying typical *FXN* alleles with 10 GAA repeats used as the assay control. Data shown are for individual CpG residues from 100 randomly sorted DNA reads arranged vertically from most to least methylated per genomic DNA sample. Summary panel below shows average methylation at individual CpGs in the *FXN* promoter region. **(C)** Bisulfite sequencing at individual CpGs was averaged to compare overall methylation in FRDA MSH2 WT and FRDA MSH2 KO conditions over 2 months post MSH2 knockout. Data shown is from an average of two cell lines each for FRDA MSH2 WT and FRDA MSH2 KO and error bars represent standard deviation. n.s., not significant. **(D)** 17 CpG residues from 100 individual DNA reads from bisulfite sequencing arranged vertically from most to least methylated per DNA sample. Summary panels below show the comparison of average methylation per CpG over time in each cell line

Next, we used bisulfite sequencing to assay the 718 bp region from − 816 to -98 bp upstream of the GAA repeat in intron 1 of *FXN*, which contains 17 CpG sites (Fig. 6A), a CpG density about 4 times lower than that of *FMR1* (Figure S4A). The parental FRDA iPSCs showed high methylation at all 17 CpGs, with a higher average methylation at CpGs closer to the repeat. In comparison, an iPSC line from an individual with 10 GAA repeats showed differentially demethylated residues for CpGs 1–5, 8–9, 13 and 15 (Fig. 6B), which includes 3 CpGs (5, 8 and 15) previously found to be unmethylated in typical alleles compared to FRDA alleles in lymphoblastoid cells [22]. We observed that these additional residues are also hypermethylated in FRDA iPSCs when compared to iPSCs with typical *FXN* alleles with 10 GAA repeats (Fig. 6B). A similar pattern of differential methylation was previously seen in peripheral blood mononuclear cells [23]. In the FRDA MSH2 WT lines, this region was highly methylated at each individual CpG (> 90%) and methylation did not change significantly over time (Fig. 6C). Similarly, in FRDA MSH2 KO lines, methylation in this region remained at > 90% after two months post-MSH2 knockout and there was no significant difference in the DNA methylation levels between the FRDA MSH2 WT and FRDA MSH2 KO lines (*p* = 0.72, Fig. 6C). No specific CpG residues that lost methylation over time in FRDA MSH2 KO iPSC lines were observed (Fig. 6D and Figure S6). Differential methylation analysis between MSH2 WT and MSH2 KO cells revealed no significant changes in methylation levels over time at CpG sites (adj. p-value > 0.05, Table S2). We did note that methylation was denser in the region closer to the GAA repeats, whereas CpG residues further from the repeats (CpGs #1–5) were slightly less methylated on average in both FRDA MSH2 WT and FRDA MSH2 KO cell lines (Fig. 6B and D).

### MSH2’s role in *de Novo* methylation of *FMR1* alleles is unclear

While *MSH2* transgene re-expression in DM1 MSH2 knockdown cell lines rescued CTG repeat instability, it failed to increase DNA methylation even when the repeat number exceeded the original, indicating that MSH2 was not involved in *de novo* methylation at the *DMPK* locus [55]. Our observations for the *FMR1* locus in MSH2 KO FXS ESCs and the *FXN* locus in MSH2 KO FRDA iPSCs clearly indicate that MSH2 is not required for the maintenance of DNA methylation at these regions. However, since we did not see a decrease in DNA methylation with MSH2 KO, we could not use the *MSH2* transgenic re-expression strategy to study its role in *de novo* methylation in either FRDA iPSCs or FXS ESCs.

**Fig. 7:**
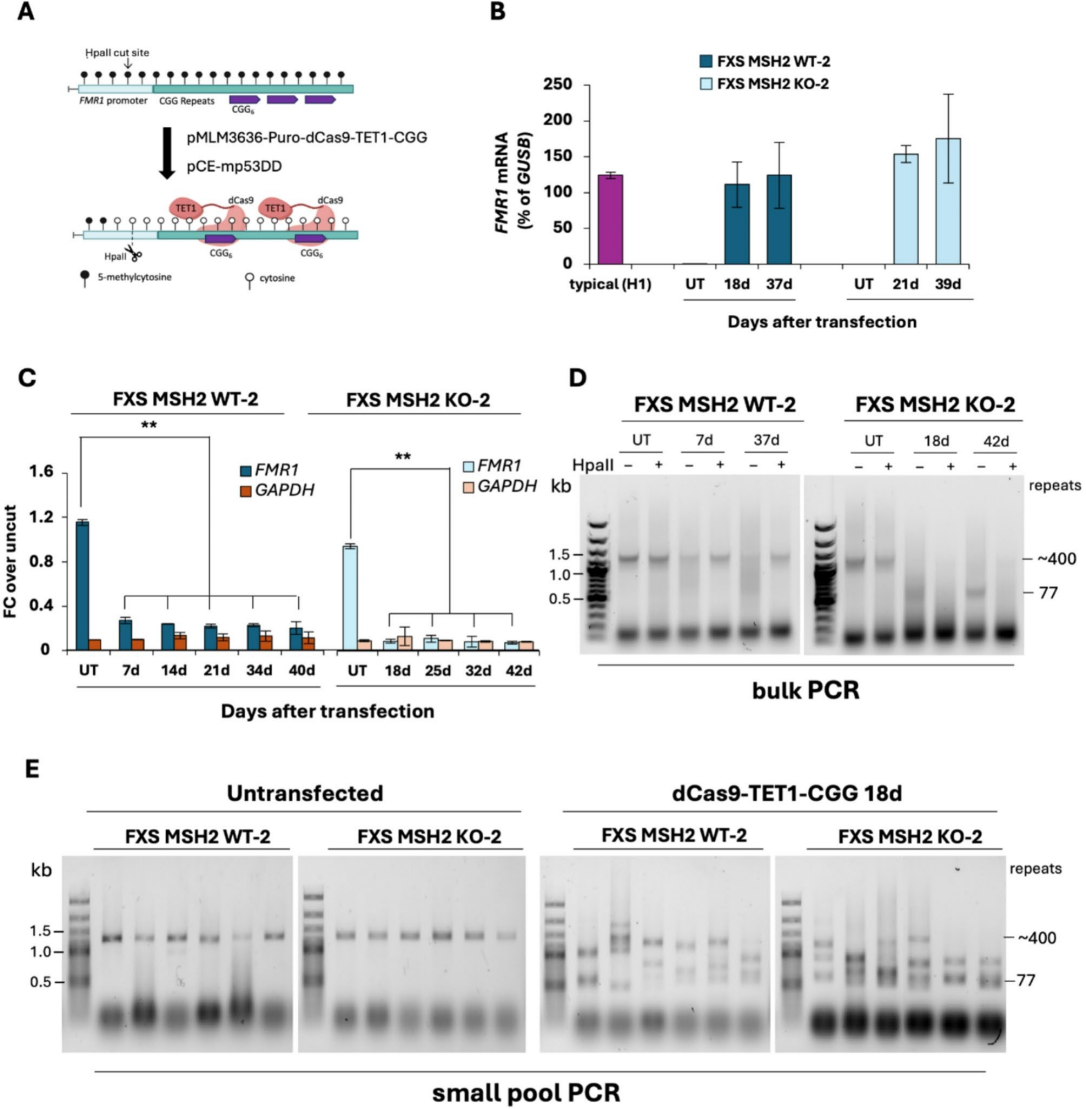
Transient transfection of FXS MSH2 WT-2 and FXS MSH2 KO-2 cell lines with plasmid pMLM3636-Puro-dCas9-TET1-CGG (dCas9-TET1-CGG). **(A)** A schematic showing the transient transfection of FXS MSH2 WT-2 and FXS MSH2 KO-2 cell lines with pMLM3636-Puro-dCas9-TET1-CGG, resulting in demethylation of the repeat region. **(B)***FMR1* mRNA levels are shown for FXS MSH2 WT-2 and FXS MSH2 KO-2 cells untransfected (UT) and at different days post transfection with plasmid dCas9-TET1-CGG. Data shown are an average of two independent experiments and error bars represent standard deviation. **(C)** Methylation-specific qPCR results for the *FMR1* promoter after dCas9-TET1-CGG transfection in FXS MSH2 WT-2 and FXS MSH2 KO-2 cells. *GAPDH* is used as an unmethylated control for HpaII digestion. Data shown are an average of two independent experiments and error bars represent standard deviation. ** *p* = 0.001. **(D)** Bulk CGG repeat PCR on DNA from transfection replicate 1 (T1) in FXS MSH2 WT-2 and FXS MSH2 KO-2 cells with plasmid dCas9-TET1-CGG. HpaII digested DNA lanes show amplification of alleles that are fully methylated while undigested samples amplify both methylated and unmethylated alleles. The *FMR1* gene in FXS ESCs carries 400 repeats resulting in a PCR product of about 1.4 kb. FXS MSH2 WT-2 and FXS MSH2 KO-2 cells show CGG repeat contractions indicated by discrete bands and smears below 1 kb. FXS MSH2 KO cells show almost complete loss of methylated full-length alleles. Bulk CGG PCR results from transfection replicate 2 (T2) are shown in Figure S8. **(E)** Small pool PCR on the DNA sample from transfection replicate 1 (T1) shows full length PCR product in untransfected (UT) cells and contracted alleles in both FXS MSH2 WT-2 and FXS MSH2 KO-2 cells transfected with plasmid dCas9-TET1-CGG

Previous work has shown that the *FMR1* gene can be transiently demethylated and reactivated in FXS lymphoblastoid and fibroblast cells by treatment with 5-azadeoxycytidine (AZA), and the gene is re-silenced in approximately three weeks after removal of AZA [35, 82]. We have previously used this strategy to study the role of the PRC2 complex in *FMR1* gene silencing [83]. However, given the toxicity of AZA treatment, we could not use this strategy to evaluate the role of MSH2 in *de novo* DNA methylation of *FMR1* alleles in FXS ESCs. We therefore used transient expression of ten-eleven translocation 1 (TET1) to reactivate the gene. TET1 catalyzes the first step in the demethylation of 5mC in CpGs [84, 85] and has been previously used to reactivate the *FMR1* gene in FXS cells [51, 86, 87]. In one of these studies [51], massive contractions of CGG repeats were observed 27 days after lentivirus-mediated expression and targeting of TET1 to CGG repeats in FXS ESCs. However, these contractions were reported to be dependent on MSH2 [51]. We reasoned that given the requirement of MSH2 for repeat contractions in FXS ESCs, transient plasmid-derived expression of TET1 in MSH2 KO FXS ESCs might allow us to reactivate the gene without generating contractions. Therefore, we transiently transfected FXS MSH2 WT-2 and FXS MSH2 KO-2 cell lines with a plasmid expressing both dCas9-TET1 and a guide RNA targeting the CGG repeats (gRNA-CGG) (Fig. 7A). We measured *FMR1* mRNA levels by RT-qPCR and DNA methylation by methylation-specific qPCR over 40 days in culture. However, because of the reduced survival of transfected cells after puromycin selection (likely due to a low efficiency of plasmid transfection), the earliest time point at which we could collect the samples was at day 7 for FXS MSH2 WT cells and day 18 for FXS MSH2 KO cells. We observed the expected increase in *FMR1* mRNA (Fig. 7B) and decrease in DNA methylation (Fig. 7C) in both FXS MSH2 WT and FXS MSH2 KO cells; however, the gene remained unmethylated and active for more than 37 days in culture expressing *FMR1* mRNA at levels similar to or higher than that observed in H1 ESCs carrying *FMR1* alleles with the typical number of CGG repeats. This was not due to the presence of residual dCas9-TET1 plasmid (Figure S7). Rather, repeat size analysis showed that in both MSH2 WT-2 and MSH2 KO-2 cell lines, the band corresponding to the 400-CGG repeat was almost completely gone, replaced by a smear of unmethylated alleles with a wide range of repeat contractions (Fig. 7D and Figure S8). Digestion of the template prior to PCR using the methylation-sensitive enzyme HpaII which has recognition sites within the PCR amplicon revealed the presence of fully methylated large alleles in some samples. However, these were such a small proportion of the total allele population that they were not amplified unless the unmethylated alleles were digested and thus could not serve as a PCR template. The absence of a full-length PCR product was not due to the poor quality of genomic DNA as the small pool PCR on the same DNA sample showed contracted alleles of similar sizes in both MSH2 WT and MSH2 KO cells (Fig. 7E).

**Fig. 8:**
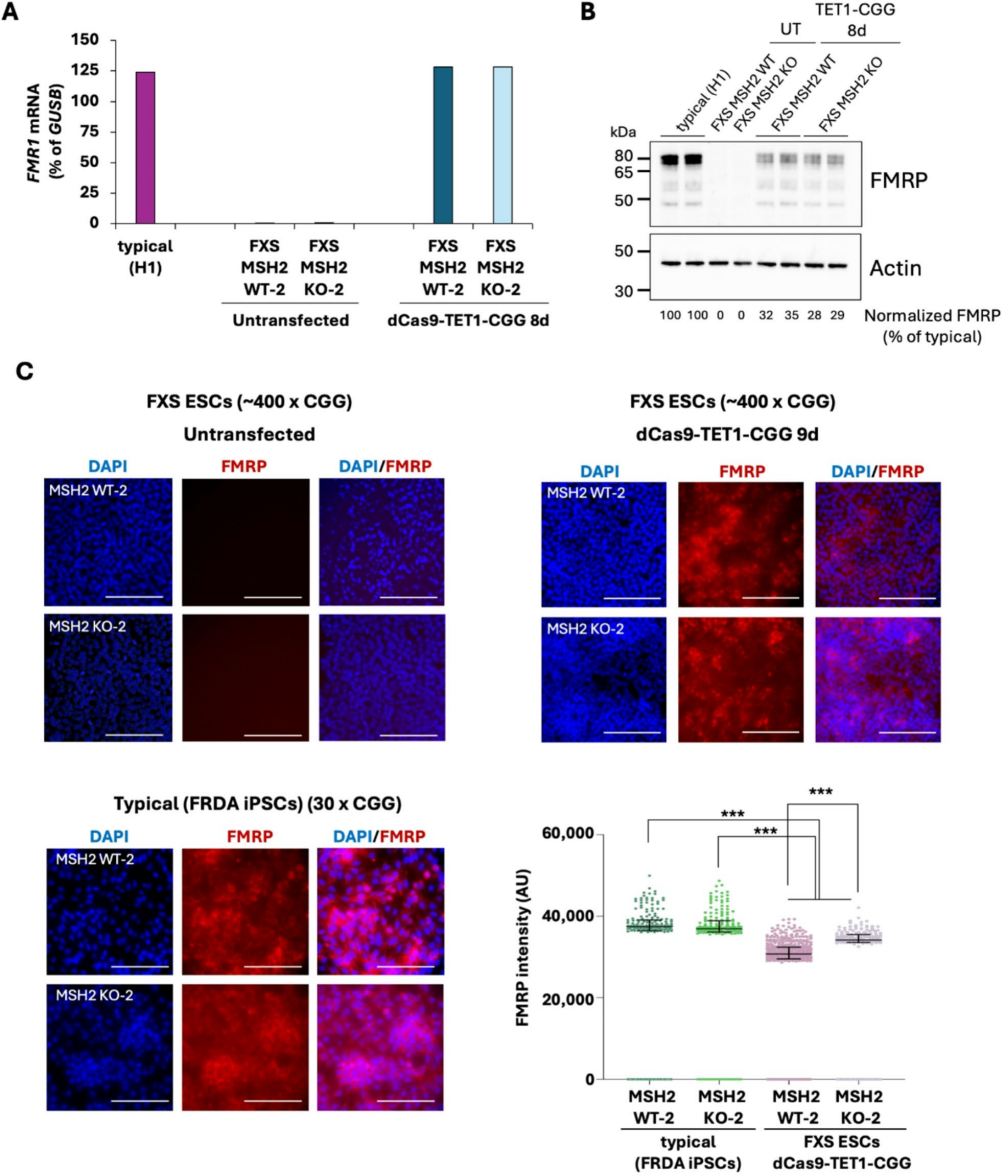
**(A)***FMR1* mRNA levels are shown for FXS MSH2 WT-2 and FXS MSH2 KO-2 cells, untransfected (UT) and 8d post transfection with plasmid pMLM3636-Puro-dCas9-TET1-CGG (dCas9-TET1-CGG). **(B)** Western blot showing FMRP expression in H1 ESCs with a typical *FMR1* allele with 30 CGG repeats and FXS MSH2 WT-2 and FXS MSH2 KO-2 cells untransfected and 8d post-transfection with dCas9-TET1-CGG. β-actin was used as a loading control for normalization and FMRP expression was calculated as % of typical (H1). **(C)** Immunofluorescence for FMRP (red) shows cytoplasmic signal in both FXS MSH2 WT-2 and FXS MSH2 KO-2 cells after transient transfection with dCas9-TET1-CGG. Scale bars represent 150 μm. Scatter dot plot shows the median with interquartile range of FMRP signal intensity (AU, arbitrary units) in MSH2 WT and MSH2 KO FRDA iPSCs (typical *FMR1* alleles with 30 CGGs) and MSH2 WT and MSH2 KO FXS ESCs transfected with dCas9-TET1-CGG. ***p < 0.0001

To confirm that the contracted alleles expressed FMRP, we transfected FXS MSH2 WT-2 and FXS KO-2 cells with dCas9-TET1 and a guide RNA targeting the CGG repeats (gRNA-CGG) and collected samples at day 8 post-transfection. We observed similar CGG repeat contractions (Figure S9A), similar levels of *FMR1* mRNA (Fig. 8A) and similar reduction in DNA methylation (Figure S9B), as observed earlier, in both FXS MSH2 WT and FXS MSH2 KO cells transfected with dCas9-TET1 and gRNA-CGG. Western blot analysis showed that the overall FMRP levels in both MSH2 WT and MSH2 KO transfected FXS cells (with repeat contractions) were ~ 30% of that seen in H1 cells with typical *FMR1* alleles with 30 CGG repeats (Fig. 8B) and immunofluorescence analysis showed that while 80% of the transfected FXS MSH2 WT and FXS MSH2 KO cells expressed FMRP, there were significant differences in the intensity of FMRP expression between FRDA iPSCs (carrying typical *FMR1* alleles) and FXS ESCs transfected with dCas9-TET1-CGG (*p* < 0.0001) and between FXS MSH2 WT and FXS MSH2 KO cells transfected with dCas9-TET1-CGG (*p* < 0.0001) (Fig. 8C).

**Fig. 9:**
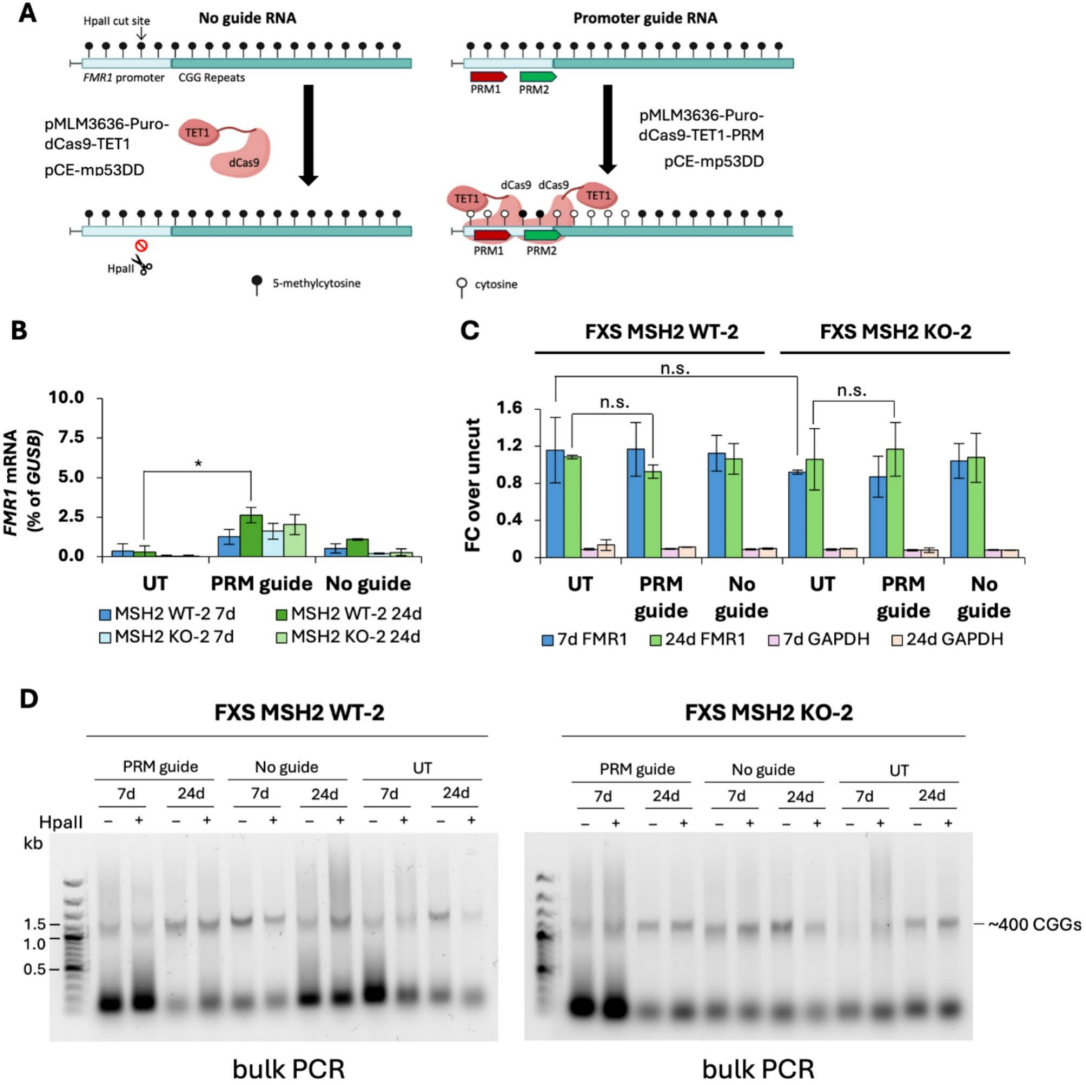
Transient transfection of FXS MSH2 WT-2 and FXS MSH2 KO-2 cell lines with plasmid pMLM3636-Puro-dCas9-TET1 and pMLM3636-Puro-dCas9-TET1-PRM. **(A)** A schematic showing the transient transfection of FXS MSH2 WT-2 and FXS MSH2 KO-2 cell lines with plasmid pMLM3636-Puro-dCas9-TET1 with no guide RNA or with plasmid dCas9-TET1-PRM with 2 gRNAs directed to the *FMR1* promoter. **(B)***FMR1* mRNA levels in FXS MSH2 WT-2 and FXS MSH2 KO-2 cells are shown as a percentage of *GUSB* mRNA in untransfected (UT) cells and at day 7 (7d) and day 24 (24d) after transient transfections. Data shown are an average of two independent experiments and error bars represent standard deviation. * *p* = 0.04. **(C)** Methylation-specific qPCR for the *FMR1* promoter at day 7 (7d) and day 24 (24d) after transient transfection with plasmids expressing dCas9-TET1 and gRNAs targeted to the *FMR1* promoter or dCas9-TET1 with no guide. *GAPDH* is used as an unmethylated control for HpaII digestion. Data shown are an average of two independent experiments and error bars represent standard deviation. n.s., not significant. **(D)** Bulk CGG repeat PCR on DNAs from transfection replicate 1 (T1) at 7d and 24d for cells transfected with plasmid dCas9-TET1-PRM (PRM guide) and plasmid dCas9-TET1 without any gRNAs (No guide) and on DNA from untransfected FXS ESCs (UT). HpaII-digested DNA lanes show amplification of alleles that are fully methylated while undigested samples amplify both methylated and unmethylated alleles. The *FMR1* gene in FXS ESCs carries 400 repeats resulting in a PCR product of about 1.4 kb. FXS MSH2 WT and FXS MSH2 KO cell lines transfected with dCas9-TET1-PRM and dCas9-TET1 with no guide FXS MSH2 WT and FXS MSH2 KO cell lines transfected with dCas9-TET1-PRM and dCas9-TET1 with no guide show CGG repeat sizes similar to untransfected (UT) cells at day 7 (7d) and day 24 (24d). The results of bulk CGG repeat PCR for a replicate transfection (T2) are shown in Figure S10A. Small pool PCR results for FXS MSH2 WT and FXS MSH2 KO cell lines transfected with dCas9-TET1-PRM at 24d are shown in Figure S10B

Transfection with dCas9-TET1 either alone or with a dual guide targeting the *FMR1* promoter (gRNA-PRM-1 and gRNA-PRM-2) (Fig. 9A) led to very low levels of *FMR1* mRNA in both FXS MSH2 WT-2 and FXS MSH2 KO-2 cell lines (Fig. 9B), likely due to insufficient TET1-targeting as evidenced by a lack of demethylation of the *FMR1* promoter (Fig. 9C). There were no significant changes seen in the repeat size upon transfection with dCas9-TET1 either alone or with gRNAs targeting the *FMR1* promoter in both bulk and small pool PCR (Fig. 9D and Figure S10).

## Discussion

To test whether the role of MSH2 in the maintenance of DNA methylation seen at the *DMPK* locus in DM1 ESCs was conserved in two other REDs, FXS and FRDA, we made null mutations in *MSH2* in FXS ESCs and FRDA iPSCs and examined the effect on DNA methylation upstream of the expanded repeats for *FMR1* and *FXN* alleles respectively. The DNA methylation at the *FMR1* locus was more variable in the FXS ESCs compared to the DNA methylation at the *FXN* locus in FRDA iPSCs at all time points in both cells expressing MSH2 and those that did not. The region upstream of the GAA repeats in the *FXN* gene has three interspersed repeated DNA elements, namely, a member of the MIRb family, a member of the primate-specific MERI family and the first half of an Alu element [22]. These elements are frequently hypermethylated [88] and thus may contribute to the more homogeneous nature of CpG methylation in FRDA cells. It may also be that the difference in the stability of DNA methylation reflects differences between ESCs and iPSCs. Despite the intrinsic variability of the FXS alleles, there was no statistically significant difference in the extent of CpG methylation of these alleles in MSH2 WT and MSH2 KO cells over 3 months in culture. It is formally possible that although ESCs divide every 24 h, given the high CpG density in FXS ESCs it would take more than 90 cell divisions for any passive demethylation to become apparent. However, the failure to see an effect of the loss of MSH2 at FRDA alleles suggests that there is more than one mechanism involved in the maintenance of DNA methylation upstream of the expanded repeats, with the MSH2-independent process being responsible for the gene silencing in FXS and FRDA that causes disease pathology. The MSH2-independent methylation process involved may be analogous to that observed for the region downstream of the expanded CTG repeats in DM1 ESCs [55].

Unfortunately, we could not use the *MSH2* transgene re-expression strategy to evaluate the role of MSH2 in *de novo* methylation in FXS and FRDA cells as we did not observe a decrease in DNA methylation upon MSH2 knockout. Furthermore, we were also unable to test the effect of MSH2 on *de nov*o methylation in FXS cells after TET1 mediated demethylation of the *FMR1* promoter because, in contrast to a previous report [51], reactivation of FXS alleles resulted in a high frequency of contractions even in cells lacking MSH2 (Fig. 7E). The contractions in both MSH2 WT and MSH2 KO lines gave rise to *FMR1* alleles with repeat sizes that were below the threshold for *de novo* methylation [14, 69, 89]. We could also not use the same demethylation strategy of targeting dCas9-TET1 to the *FXN* locus in FRDA cells because the GAA repeat itself cannot be methylated as it lacks CpG sites; it is not a PAM site for SpCas9 targeting and, as we observed for the *FMR1* locus, targeting dCas9-TET1 using one or two gRNAs to individual CpG sites outside the repeats would not result in appreciable demethylation.

While targeting TET1 to the FX repeat had a large effect on gene reactivation, very little effect was seen when TET1 was overexpressed alone or when it was targeted to the *FMR1* promoter region (Fig. 9B and C), and no repeat contractions were seen in either FXS MSH2 WT-2 or FXS MSH2 KO-2 cells (Fig. 9D and Figure S10). This suggests that TET1 activity directed to the CGG repeats is necessary for demethylation of the entire *FMR1* promoter region, gene reactivation and CGG repeat contractions. This effect may be related to competition between dCas9 and DNMT1 for binding to the locus [87] or due to repeat contractions resulting from either the processing of R-loops induced by binding of multiple gRNAs targeting CGGs [51] or via the consequences of replication fork stalling by multiple dCas9 molecules bound to the repeats.

An unintended consequence of our attempts to study MSH2’s role in *de novo* methylation was the finding that transcription drives at least two types of CGG repeat contractions, with a significant number of these contractions being MSH2-independent. MSH2-dependent contractions have previously been observed on reactivation of FX alleles [51]. Because MSH2 binding stabilizes MSH3 and MSH6 and the loss of MSH2 drastically reduces the levels of both MSH3 and MSH6 [55, 90], we cannot definitively attribute these MSH2-dependent contractions to MutSβ, although given the preferential binding of MutSβ to the intrastrand structures formed by FX repeat, and the demonstration than MSH3-dependent contractions are seen in a FXD mouse model [54], a role for MutSβ in the MSH2-dependent contractions seems likely.

We and others have shown that actively transcribed *FMR1* alleles form R-loops that become more stable with increasing repeat number [83, 91, 92]. R-loops are a frequent source of DNA damage, in part because the single-stranded DNA associated with the R-loop is prone to a variety of types of DNA damage even in the absence of replication [93–95]. Furthermore, stable R-loops are also a frequent source of transcription-replication collisions. As such the R-loops formed on transcribed FX alleles may represent source of significant transcriptional stress that results in the contractions seen on reactivation of silenced FX alleles. A transcription-coupled repair (TCR) process has been implicated in CAG-repeat contractions in a reporter construct in human cells [96]. This process is MSH2-dependent and may account for those contractions that require MSH2. In yeast, CAG-repeats have been shown to undergo transcription-induced contractions via an MSH2-independent process [97]. Whether a similar mechanism is responsible for the MSH2-independent CGG-contractions we observe remains to be seen. A better understanding of the mechanisms underlying transcription induced repeat contractions may allow these processes to be harnessed to safely drive alleles from above a pathological repeat size threshold into or closer to the typical repeat size. The fact that transcription drives these contractions so effectively lends support to the idea that transcriptional silencing may be a cellular response to limit the risk to genome integrity.

## Conclusions

We have demonstrated that loss of MSH2 does not affect DNA methylation at the *FMR1* promoter in FXS ESCs or in intron 1 of the *FXN* gene in FRDA iPSCs. While we could not evaluate the role of MSH2 in establishing DNA methylation at the *FMR1* locus in FXS ESCs, our results clearly show that MSH2 is not required for the maintenance of DNA methylation induced by repeat expansion in either FXS or FRDA, and that this difference from what is seen in DM1 cannot be attributed to the difference in the overall GC content or CpG density of the regions examined. We suggest that any difference in the requirement of factors in the establishment or maintenance of repeat induced epigenetic changes may be due to the differences in the sequence or the structures formed by the expanded repeats. More work is needed to understand what these differences might be. Our work also revealed the existence of an MSH2-independent pathway for transcription-induced contractions. Both MSH2-dependent and MSH2-independent contractions may contribute to the repeat size mosaicism seen in individuals with transcriptionally active unmethylated FX alleles that can influence the clinical presentation of the disease [98–100].

## Electronic supplementary material

Below is the link to the electronic supplementary material.

Supplementary Material 1

Supplementary Material 2

Supplementary Material 3

Supplementary Material 4

Supplementary Material 5

Supplementary Material 6

## Data Availability

All data generated in this study are included in the manuscript and supplementary material.

## Abbreviations

RED: Repeat Expansion Disorder
DM1: Myotonic Dystrophy Type 1
FXS: Fragile X Syndrome
FRDA: Friedreich’s Ataxia
UTR: Untranslated Region
MMR: Mismatch Repair
hESC: Human Embryonic Stem Cell
iPSC: Induced Pluripotent Stem Cell
