# Supplementary material for: MSH2 is not required for either maintenance of DNA methylation or repeat contraction at the FMR1 locus in fragile X syndrome or the FXN locus in Friedreich’s ataxia
Source: Epigenetics Chromatin. 2025 Apr 28;18:24. doi: 10.1186/s13072-025-00588-4 (PMC12036138; doi:10.1186/s13072-025-00588-4)
Supplement: Supplementary file 6 — Supplementary Material 6 [file 13072_2025_588_MOESM6_ESM.docx]

**Table S1**: Primer and Guide RNA Sequences

| **ID #** | **Primer Name** | **Primer Sequence (5’-3’)** |
| --- | --- | --- |
| 1 | pX462-MSH2-dual-F | GAAAGGACGAAACACCGGTATGTGGATTCCATACAGTTAGAGCTAGAAATA |
| 2 | pX462-MSH2-dual-R | TTTCTAGCTCTAAAACCAGTTGATGGCCAGAGACAGCGGTGTTTCGTCCTTT |
| 3 | Gb_MSH2_Ex3F | TTTGGATTTTTCCTTTTTGCTT |
| 4 | Gb_MSH2_Ex3R | CCACATGCCTATACAAATGACA |
| 5 | *FMR1* ex1 F | GAACAGCGTTGATCACGTGAC |
| 6 | *FMR1* ex1 R | GTGAAACCGAAACGGAGCTGA |
| 7 | *GAPDH* exon1 F | TCGACAGTCAGCCGCATCT |
| 8 | *GAPDH* intron1 R | CTAGCCTCCCGGGTTTCTCT |
| 9 | FMR1-Met-IF | GGAATTTTAGAGAGGTYGAATTGGG |
| 10 | Gb_metbis-2645-R | aaacgacggccagtgCTCAAAAACTACCCTCCACC |
| 11 | Gb_FMR1-Met-2F | cacatcgctcagacacGTTATTGAGTGTATTTTTGTAGAAATGGG |
| 12 | Not_FraxC | AGTTCAGCGGCCGCGCTCAGCTCCGTTTCGGTTTCACTTCCGGT |
| 13 | FAM_Not_FraxR4 | CAAGTCGCGGCCGCCTTGTAGAAAGCGCCATTGGAGCCCCGCA |
| 14 | GAA-104F | GGCTTAAACTTCCCACACGTGTT |
| 15 | GAA-629R | AGGACCATCATGGCCACACTT |
| 16 | Gb_FXNMe-1212-F | cacatcgctcagacacGAGGTGAAATTTTTAGAGTTGTAGAATAGTTAGAGTAGTAG |
| 17 | Gb_FXNMe_1930-R | aaacgacggccagtgCACCTCCCAAAATACTAAAATTATAAACATAAACCA |
| 18 | PuroR-FMR1-gRNA-PRM_F | GAAAGGACGAAACACCgacagcgttgatcacgtgacggttttagagctaGAAAtagc |
| 19 | PuroR-FMR1-gRNA-PRM_R | TTTCtagctctaaaacggtcgaaagacagacgcgcgcGGTGTTTCGTCCTTT |
| 20 | Gb_3636-TET1-F | TCACTTTTTTTCAGGTTGGATACCCTCGTAAAGGCCACC |
| 21 | Gb_3636-TET1-R | TGTACTCGGTCATGGTGGCACCAGGGCCGGGATTCTCCTC |
| 22 | Gb_dCas9-cmyc-F | CACTTCCTACCCTCGTAAAGGCCACCATGGGACCAGCCGCAAAGAGAGTGAAACTGGACGGAGGTCCTGCTGCAAAAAGGGTGAAGTTGGATGGAGACTACAAAGACCATGACGGTG |
| 23 | gRNA-CGG_6_ | acaccGGCGGCGGCGGCGGCGGCGGg |
| 24 | gRNA-PRM-1 | ACAGCGTTGATCACGTGACG |
| 25 | gRNA-PRM-2 | CGCGCGTCTGTCTTTCGACC |
| 26 | 3636-727F | AAGCAGGCTTTAAAGGAACCA |
| 27 | 459-377R | TACCCGTTACATAACTTACG |

**Table S2**: Differential methylation analysis between MSH2 WT and MSH2 KO cells over time at CpG sites in the *FMR1* gene in FXS and *FXN* gene in FRDA cells

| **Gene** | **CpG site** | **Genotype:Month interaction** | |
| --- | --- | --- | --- |
|  |  |  | |
|  |  | **p.value** | **adj.p.value** |
| *FMR1* | CpG 1 | 0.0543 | 1 |
| *FMR1* | CpG 2 | 0.6691 | 1 |
| *FMR1* | CpG 3 | 0.3977 | 1 |
| *FMR1* | CpG 4 | 0.3932 | 1 |
| *FMR1* | CpG 5 | 0.2725 | 1 |
| *FMR1* | CpG 6 | 0.2434 | 1 |
| *FMR1* | CpG 7 | 0.0462 | 1 |
| *FMR1* | CpG 8 | 0.4918 | 1 |
| *FMR1* | CpG 9 | 0.6297 | 1 |
| *FMR1* | CpG 10 | 0.4095 | 1 |
| *FMR1* | CpG 11 | 0.6092 | 1 |
| *FMR1* | CpG 12 | 0.4995 | 1 |
| *FMR1* | CpG 13 | 0.4317 | 1 |
| *FMR1* | CpG 14 | 0.8044 | 1 |
| *FMR1* | CpG 15 | 0.3635 | 1 |
| *FMR1* | CpG 16 | 0.3664 | 1 |
| *FMR1* | CpG 17 | 0.3334 | 1 |
| *FMR1* | CpG 18 | 0.8101 | 1 |
| *FMR1* | CpG 19 | 0.9144 | 1 |
| *FMR1* | CpG 20 | 0.5753 | 1 |
| *FMR1* | CpG 21 | 0.9478 | 1 |
| *FMR1* | CpG 22 | 0.5044 | 1 |
| *FMR1* | CpG 23 | 0.4314 | 1 |
| *FMR1* | CpG 24 | 0.1787 | 1 |
| *FMR1* | CpG 25 | 0.4002 | 1 |
| *FMR1* | CpG 26 | 0.3332 | 1 |
| *FMR1* | CpG 27 | 0.2099 | 1 |
| *FMR1* | CpG 28 | 0.6126 | 1 |
| *FMR1* | CpG 29 | 0.2886 | 1 |
| *FMR1* | CpG 30 | 0.0709 | 1 |
| *FMR1* | CpG 31 | 0.4573 | 1 |
| *FMR1* | CpG 32 | 0.4318 | 1 |
| *FMR1* | CpG 33 | 0.1337 | 1 |
| *FMR1* | CpG 34 | 0.5347 | 1 |
| *FMR1* | CpG 35 | 0.3696 | 1 |
| *FMR1* | CpG 36 | 0.9719 | 1 |
| *FMR1* | CpG 37 | 0.4096 | 1 |
| *FMR1* | CpG 38 | 0.1763 | 1 |
| *FXN* | CpG 1 | 0.128 | 1 |
| *FXN* | CpG 2 | 0.3601 | 1 |
| *FXN* | CpG 3 | 0.5979 | 1 |
| *FXN* | CpG 4 | 0.4269 | 1 |
| *FXN* | CpG 5 | 0.7556 | 1 |
| *FXN* | CpG 6 | 0.5291 | 1 |
| *FXN* | CpG 7 | 0.4601 | 1 |
| *FXN* | CpG 8 | 0.8058 | 1 |
| *FXN* | CpG 9 | 0.3153 | 1 |
| *FXN* | CpG 10 | 0.2508 | 1 |
| *FXN* | CpG 11 | 0.6619 | 1 |
| *FXN* | CpG 12 | 0.0132 | 0.2244 |
| *FXN* | CpG 13 | 0.8835 | 1 |
| *FXN* | CpG 14 | 0.0156 | 0.2652 |
| *FXN* | CpG 15 | 0.1912 | 1 |
| *FXN* | CpG 16 | 0.6779 | 1 |
| *FXN* | CpG 17 | 0.5614 | 1 |

**Supplementary figures**


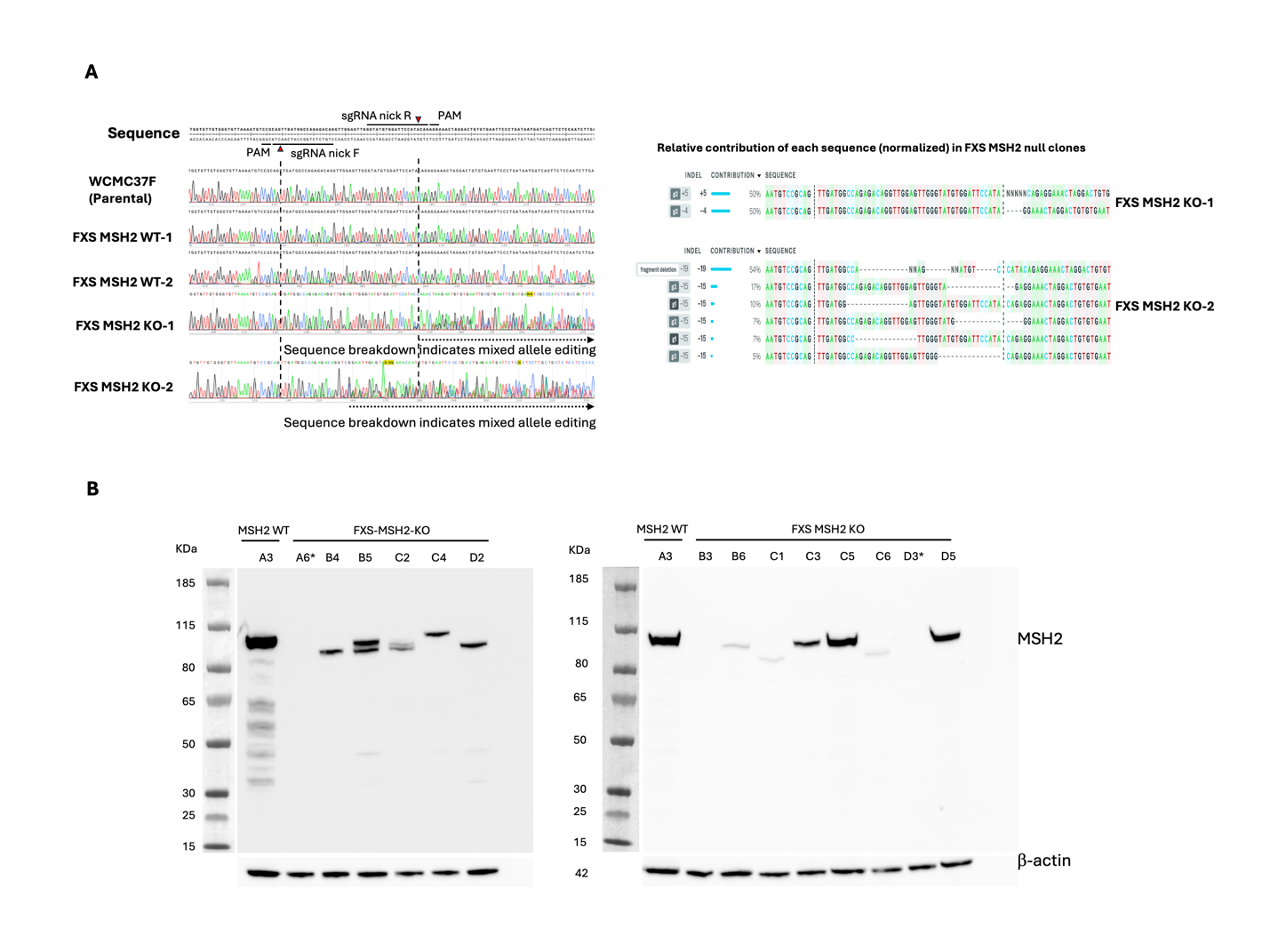


**Figure S1. (A)** Sanger Sequencing results for MSH2 exon 3 PCR on FXS MSH2 WT and FXS MSH2 KO cell lines aligned to MSH2 exon 3. Diagram shows location of nickase CRISPR guide RNAs and single strand break locations. FXS MSH2 WT samples show 100% match to reference sequence and both FXS MSH2 KO samples display sequence breakdown within the two CRISPR nick sites indicative of CRISPR editing and mixed allele population. Sequence analysis using Synthego ICE software shows complete loss of *MSH2* exon 3 allele in both cell lines, with indel regions that match the region of sequence breakdown. **(B)** Western blot for MSH2 with β-actin loading control on single-cell clones of WCMC 37F FXS ESCs edited with dual CRISPR nickase targeting *MSH2* exon 3. All clones were analyzed here, and complete knockouts A6 and D3 (marked with asterisks) were selected for further study and renamed FXS MSH2 KO-1 and FXS MSH2 KO-2.

**
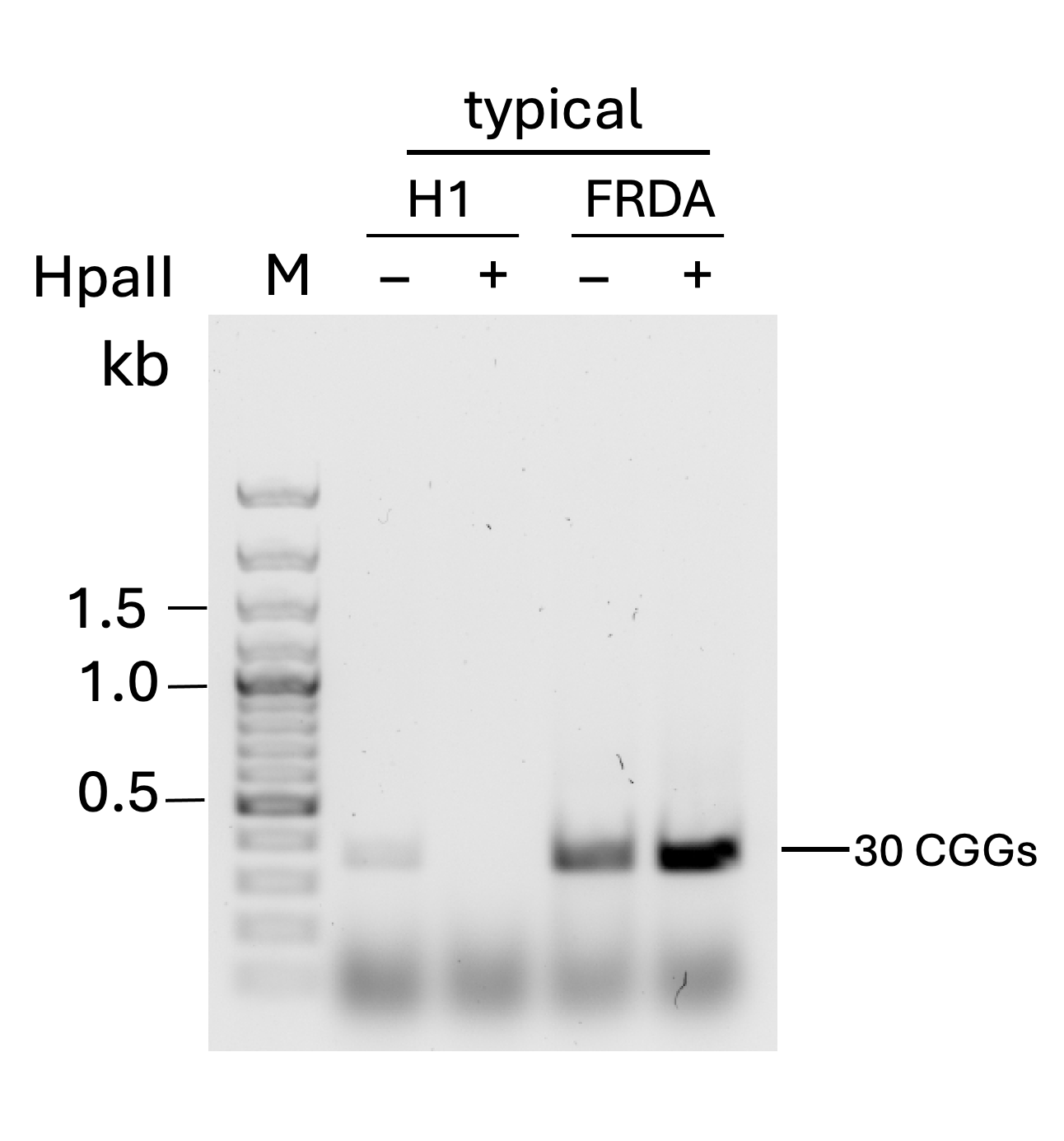
**

**Figure S2.** CGG-repeat PCR for H1 (male hESCs) and GM23404 (female FRDA iPSCs) cell lines, both carrying typical *FMR1* alleles with 30 CGG repeats. PCR on DNA digested with HpaII shows the presence of an unmethylated *FMR1* allele in H1 cells and the presence of both methylated and unmethylated *FMR1* alleles in FRDA cells due to X-inactivation.


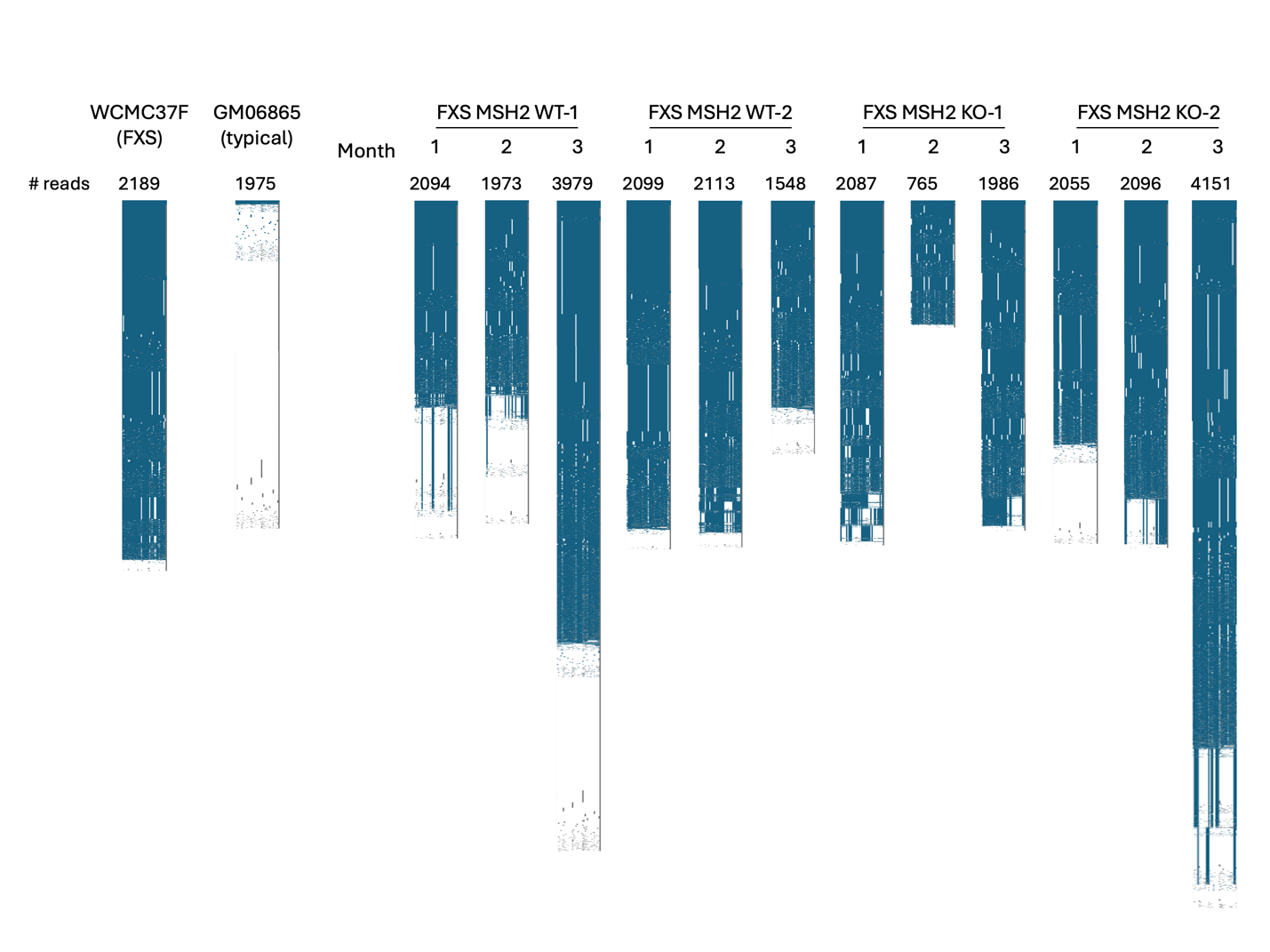


**Figure S3.** All of the bisulfite sequencing reads for *FMR1* promoter region in FXS samples from Figure 4C.


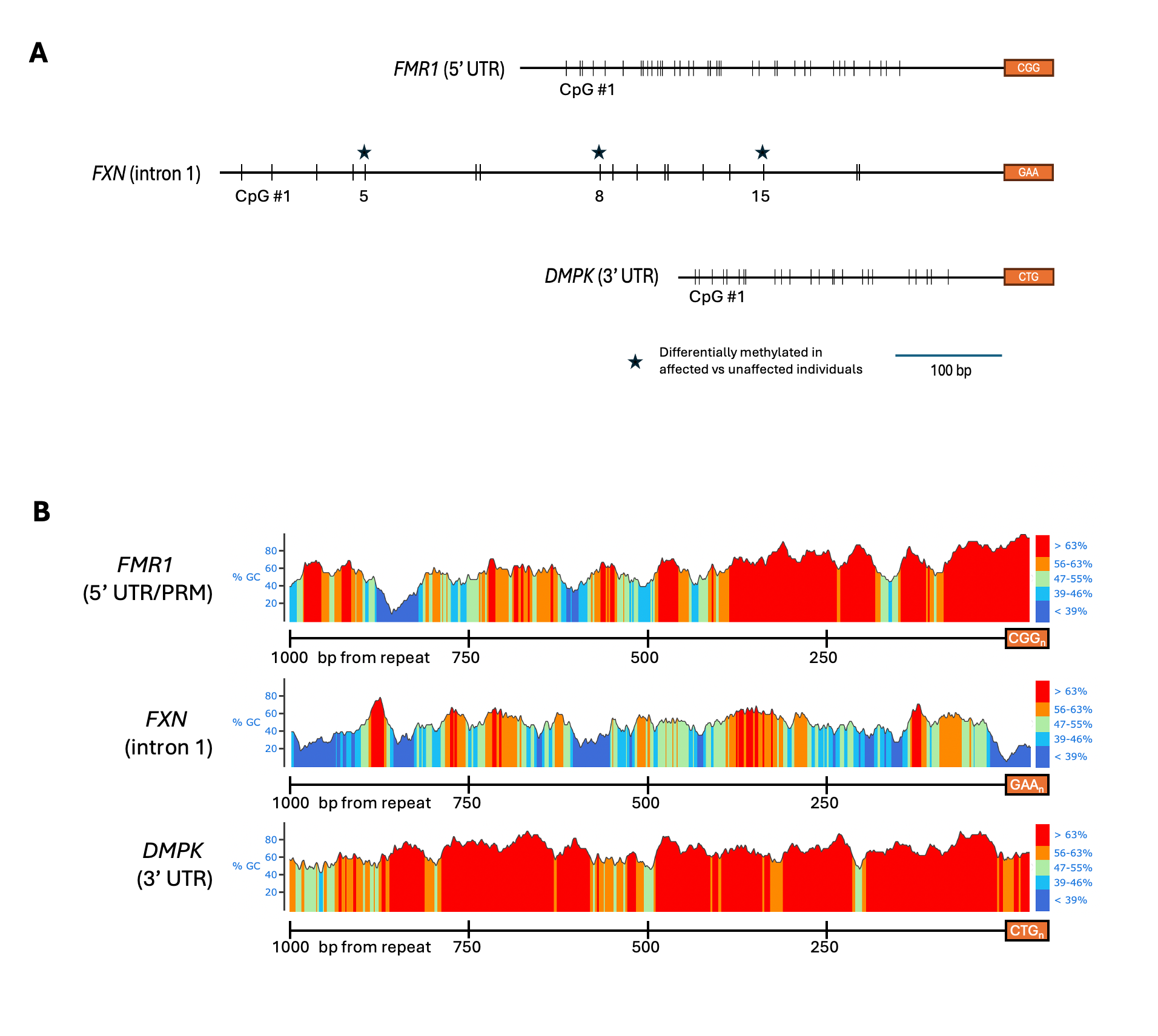


**Figure S4. (A)** Diagram showing CpG residues that are methylated in regions upstream of the disease-causing repeats in Fragile X syndrome (*FMR1),* Friedreich’s Ataxia (*FXN),* and Myotonic Dystrophy Type I (*DMPK).* CpGs that are differentially hypermethylated in FRDA vs non-FRDA lymphoblastoid cell lines (22) are noted with an asterisk and numbered. **(B)** Overall GC content of the region 1 kb upstream of the repeats in each disorder. The height of the curve and the color of the area under the curve are based on the average GC content (%) from low (blue) to high (red).

**
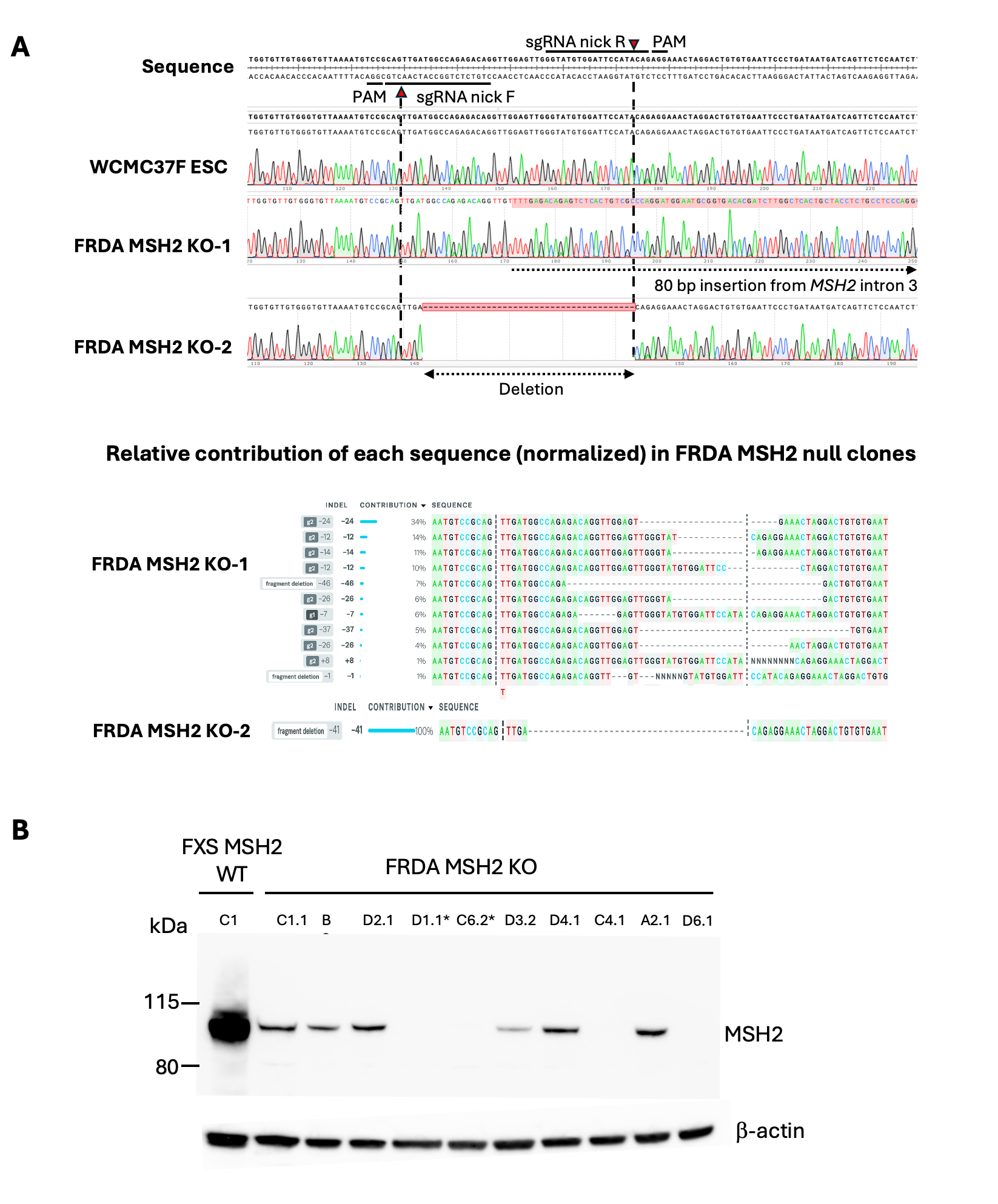
**

**Figure S5. (A)** Sanger Sequencing results for MSH2 exon 3 PCR on FRDA MSH2 KO cell lines aligned to MSH2 exon 3. Diagram shows location of nickase CRISPR guide RNAs and single strand break locations. Both FRDA MSH2 KO samples show evidence of CRISPR editing: FRDA MSH2 KO-1 has an 80 bp insertion of a region of *MSH2* intron 3 inserted in between the CRISPR cut sites and FRDA MSH2 KO-2 has a 41 bp deletion with the 3’ cut site matching the sgRNA nick R cut site. Sequence analysis using Synthego ICE software shows complete loss of *MSH2* exon 3 allele in both cell lines, with a deletion matching the Sanger sequencing alignment in FRDA MSH2 KO-2. In FRDA MSH2 KO-1, ICE did not identify the insertion, but it did confirm complete loss of the typical *MSH2* allele. **(B)** Western blot for MSH2 with β-actin loading control on single-cell clones of GM23404 FRDA human iPSCs edited with dual CRISPR nickase targeting *MSH2* exon 3. All clones isolated are analyzed here and 4 clones were found to be complete knockouts: D1.1, C6.2, C4.1, D6.1. Two clones C6.2 and D1.1 were selected for continued study (marked with asterisk in the Figure) and were renamed FRDA MSH2 KO-1 and FRDA MSH2 KO-2 respectively.


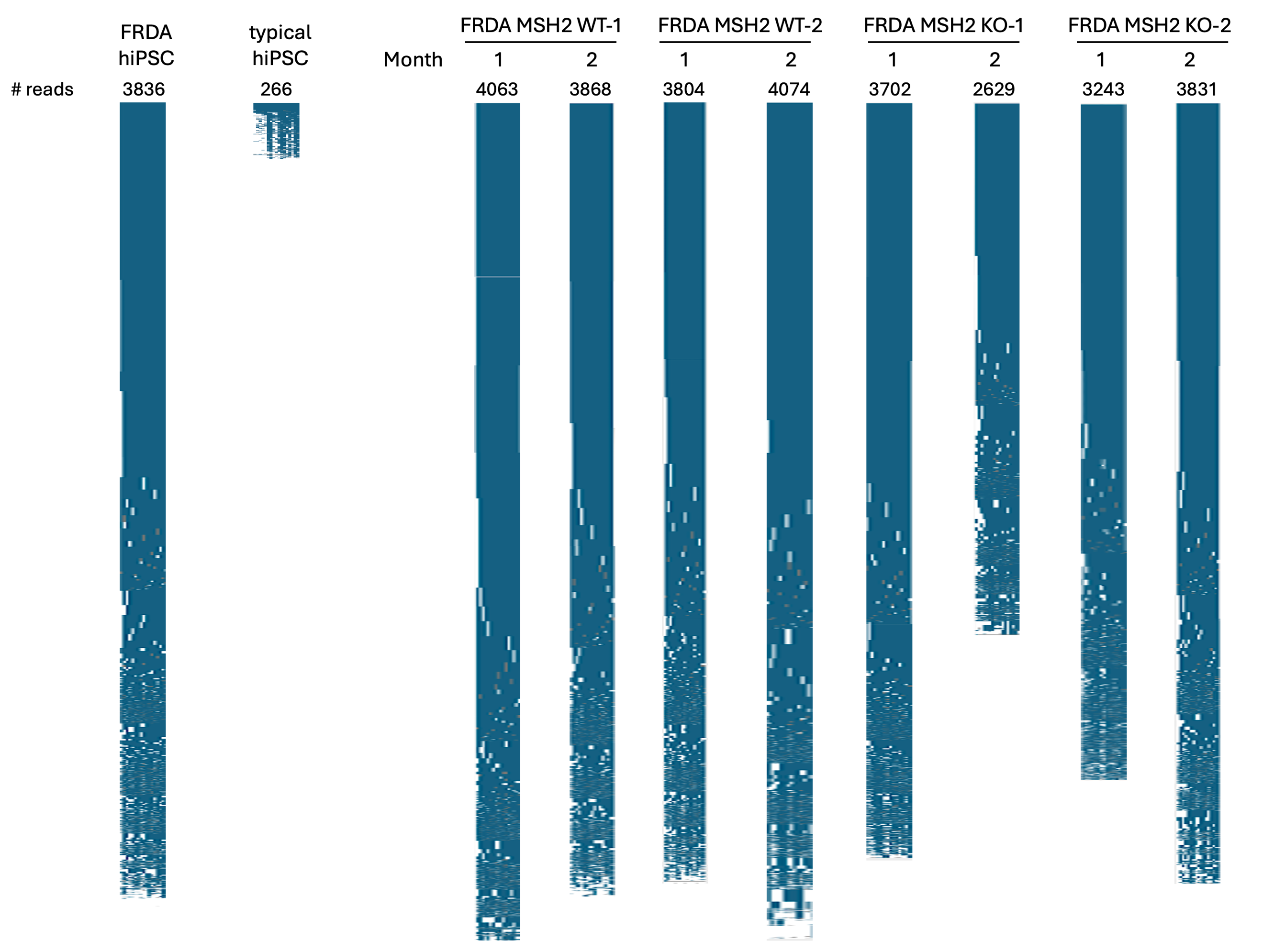


**Figure S6.** All of the bisulfite sequencing reads for *FXN* intron 1 in FRDA samples from Figure 6D.


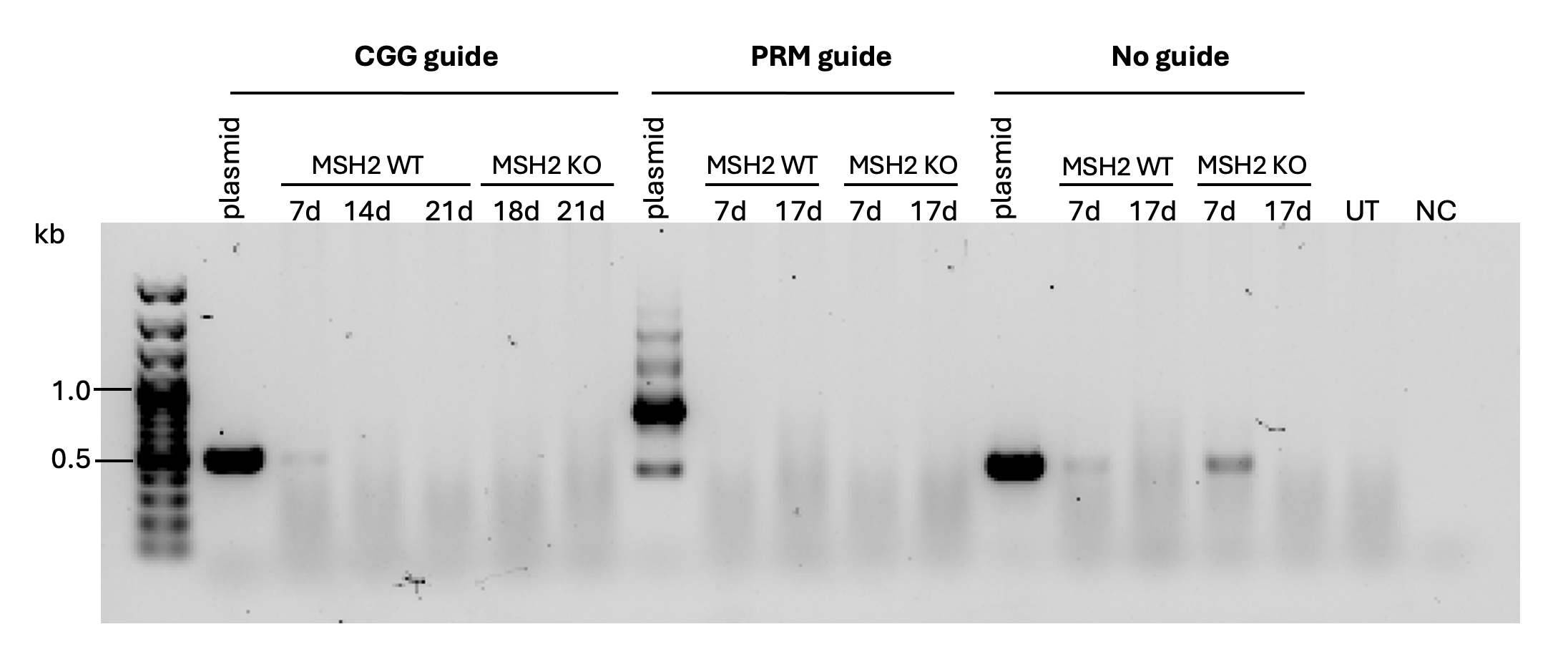


**Figure S7**. PCR showing that low levels of TET1 plasmids are present at day 7 after transfection and completely lost by 2 weeks post transfection.


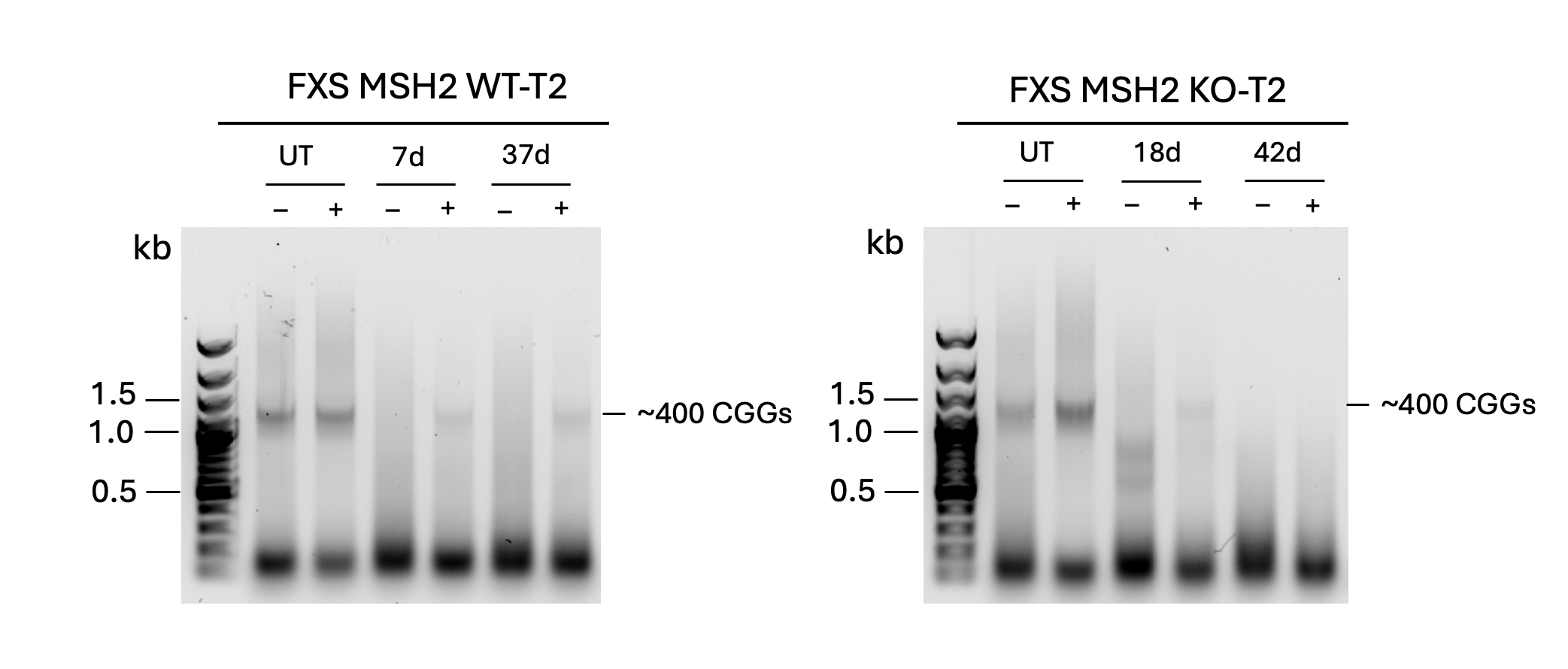


**Figure S8**. CGG repeat PCR results for DNA from transfection replicate 2 (T2) at 7d and 37d for FXS MSH2 WT-2 and 18d and 42d for FXS MSH2 KO-2 cells transfected with plasmid dCas9-TET1-CGG. HpaII-digested DNA lanes show amplification of alleles that are fully methylated while undigested samples amplify both methylated and unmethylated alleles. The *FMR1* gene in FXS ESCs carries ~400 repeats resulting in a PCR product of about 1.4 kb. The results of CGG repeat PCR for transfection replicate 1 (T1) are shown in Figure 7D.


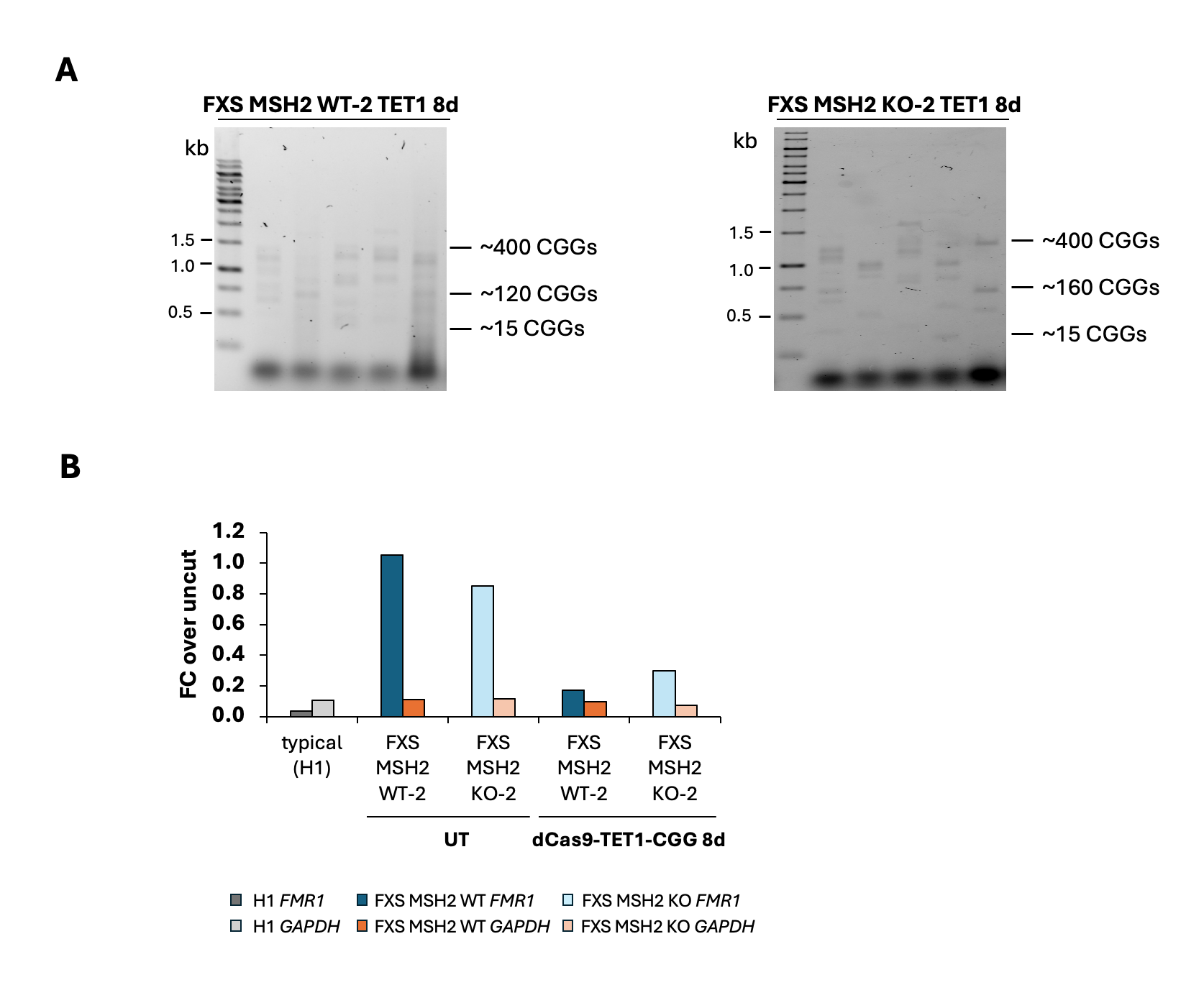


**Figure S9. (A)** Small pool PCR on samples from FXS MSH2 WT-2 and FXS MSH2 KO-2 lines collected 8d after transient transfection with dCas9-TET1-CGG_6_CG. **(B)** Methylation-specific qPCR results for the *FMR1* promoter 8d after dCas9-TET1-CGG transfection in FXS MSH2 WT-2 and FXS MSH2 KO-2 cells. *GAPDH* is used as an unmethylated control for HpaII digestion.


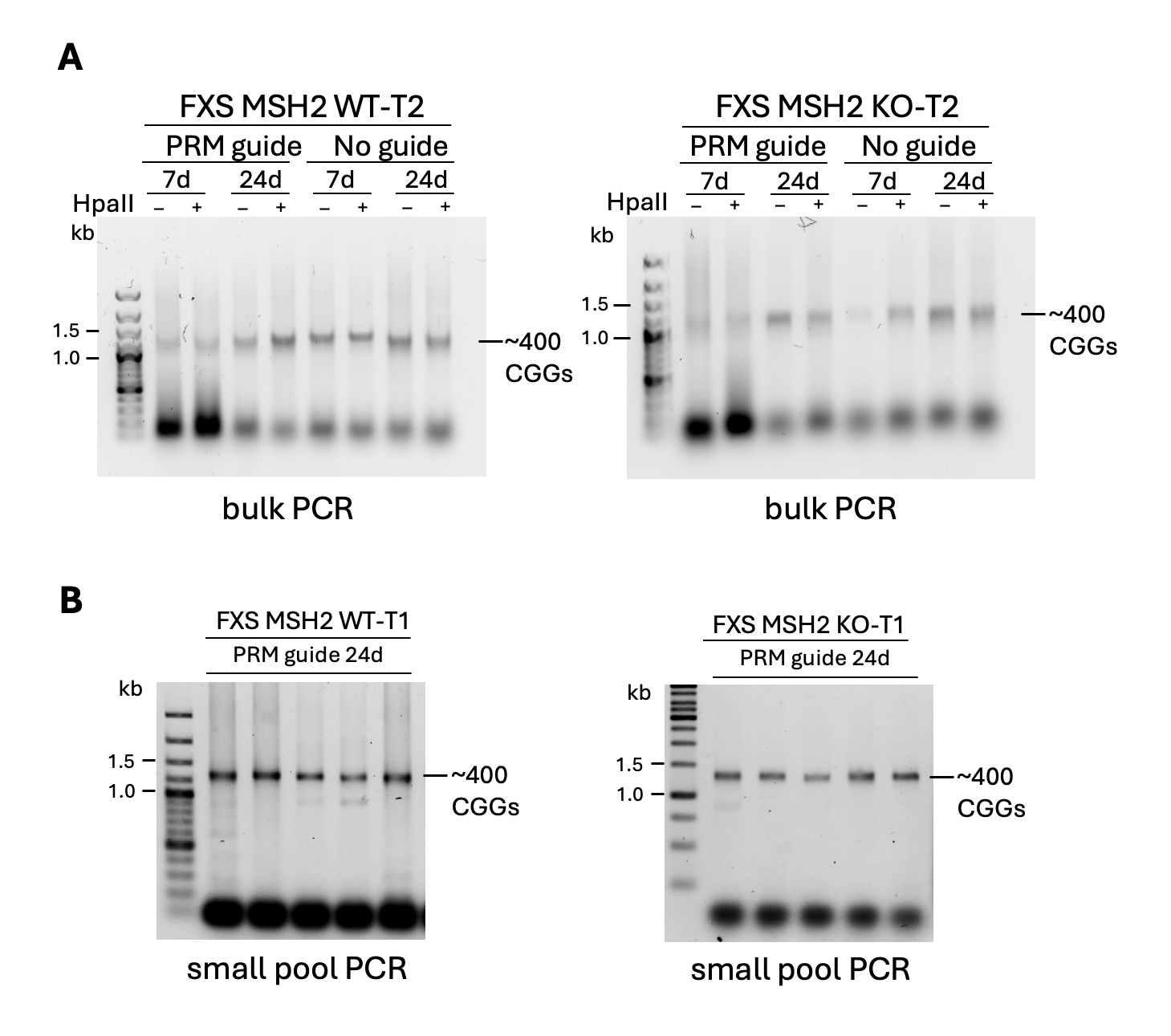


**Figure S10. (A)** Bulk CGG-repeat PCR for a second replicate of FXS MSH2 WT and FXS MSH2 KO cell lines transfected with dCas9-TET1-PRM and dCas9-TET1 with no guide.**(B)** Small pool PCR on the DNA sample from transfection replicate 1 (T1) shown in Figure 9D shows full length PCR product in both MSH2 WT and MSH2 KO cells transfected with plasmid dCas9-TET1-PRM.
